# Setting Priorities for Regional Conservation Planning in the Mediterranean Sea

**DOI:** 10.1371/journal.pone.0059038

**Published:** 2013-04-05

**Authors:** Fiorenza Micheli, Noam Levin, Sylvaine Giakoumi, Stelios Katsanevakis, Ameer Abdulla, Marta Coll, Simonetta Fraschetti, Salit Kark, Drosos Koutsoubas, Peter Mackelworth, Luigi Maiorano, Hugh P. Possingham

**Affiliations:** 1 Hopkins Marine Station, Stanford University, Pacific Grove, California, United States of America; 2 Department of Geography, The Hebrew University of Jerusalem, Mount Scopus, Jerusalem, Israel; 3 Institute of Marine Biological Resources, Hellenic Centre for Marine Research, Ag. Kosmas, Greece; 4 ARC Centre of Excellence for Environmental Decisions, School of Biological Sciences, The University of Queensland, Brisbane, Queensland, Australia; 5 European Commission, Joint Research Centre, Institute for Environment and Sustainability, Water Resources Unit, Ispra, Italy; 6 UNEP World Conservation Monitoring Centre, Cambridge, England; 7 Institute of Marine Science, Marine Renewal Resources Department (ICM-CSIC), Barcelona, Spain; 8 Laboratory of Marine Biology, Università del Salento, CoNISMa, Lecce, Italy; 9 The Biodiversity Research Group, Department of Ecology, Evolution, and Behavior, The Hebrew University of Jerusalem, Jerusalem, Israel; 10 Department of Marine Sciences, School of Environment, University of the Aegean, Mytilini, and National Marine Park of Zakynthos, Zakynthos, Greece; 11 Department of Conservation, The Blue World Institute of Marine Research and Conservation, Veli Lošinj, Croatia; 12 Department of Biology and Biotechnologies “Charles Darwin”, University of Rome “La Sapienza”, Rome, Italy; Technical University of Denmark, Denmark

## Abstract

Spatial prioritization in conservation is required to direct limited resources to where actions are most urgently needed and most likely to produce effective conservation outcomes. In an effort to advance the protection of a highly threatened hotspot of marine biodiversity, the Mediterranean Sea, multiple spatial conservation plans have been developed in recent years. Here, we review and integrate these different plans with the goal of identifying priority conservation areas that represent the current consensus among the different initiatives. A review of six existing and twelve proposed conservation initiatives highlights gaps in conservation and management planning, particularly within the southern and eastern regions of the Mediterranean and for offshore and deep sea habitats. The eighteen initiatives vary substantially in their extent (covering 0.1–58.5% of the Mediterranean Sea) and in the location of additional proposed conservation and management areas. Differences in the criteria, approaches and data used explain such variation. Despite the diversity among proposals, our analyses identified ten areas, encompassing 10% of the Mediterranean Sea, that are consistently identified among the existing proposals, with an additional 10% selected by at least five proposals. These areas represent top priorities for immediate conservation action. Despite the plethora of initiatives, major challenges face Mediterranean biodiversity and conservation. These include the need for spatial prioritization within a comprehensive framework for regional conservation planning, the acquisition of additional information from data-poor areas, species or habitats, and addressing the challenges of establishing transboundary governance and collaboration in socially, culturally and politically complex conditions. Collective prioritised action, not new conservation plans, is needed for the north, western, and high seas of the Mediterranean, while developing initial information-based plans for the south and eastern Mediterranean is an urgent requirement for true regional conservation planning.

## Introduction

Marine and terrestrial ecosystems are impacted by a suite of pressures that have led to unprecedented degradation and loss of natural habitats, and to the deterioration of ecosystem services that are essential to humanity [1]. Effective maintenance of ecosystems requires that nature conservation targets are balanced and reconciled with social, economic, cultural and political needs. It is imperative that conservation actions are carefully selected and spatially defined to yield the greatest benefits, given the constraints posed by human needs and values.

The Mediterranean Sea is a hotspot of marine diversity [2]. Of the ∼17,000 marine species reported to date in this sea approximately one fifth are considered to be endemic [3]. The Mediterranean Sea's diverse ecosystems are affected by many anthropogenic threats, some of which began thousands of years ago [4], [5], including intensifying fishing practices and resource extraction [6], [7], increasingly densely populated coastlines [6], invasive species [8], and climate change [9]. In a recent quantification of cumulative human impacts to marine ecosystems, Halpern et al. [10] found that Mediterranean marine ecoregions (*sensu* [11]) are among the twenty most impacted ecoregions of the 232 globally recognized. This pressure has resulted in major alterations of Mediterranean marine ecosystems and widespread conflict among users [4], [12], [13], [14], [15].

Currently 21 states share the Mediterranean coastline. Conservation is challenged by the inherent socio-political complexity of this region, particularly by the high diversity of political and cultural systems and legal jurisdictions [16]. While the interests of, and the relationships between, the Mediterranean States cover the entire social and political spectrum, there is recognition that the basin is a shared collective resource that is under threat [17]. The condition of these relationships is critical when considering the opportunities and obstacles for collaboration in conservation efforts among states [18].

While threats are increasing there are also unprecedented opportunities to expand the spatial scale of conservation efforts, and improve their coordination and integration throughout this region [19]. Over 100 marine protected areas (MPAs) exist in the region [13], [20], including the 84,500 Km^2^ Pelagos Sanctuary [21], with others in the planning stages. Of particular importance are networks associated with international conventions and agreements, which cover the Mediterranean wholly, or in part. The Barcelona Convention (1976) includes the Specially Protected Area Protocol (SPA Protocol, 1995), which applies to all the marine water, seabed, and terrestrial coastal areas. This protocol provides for the development of SPAs of Mediterranean Importance (SPAMIs) with clear procedures for the listing of these areas [22]. The SPAMI list represents the core of a protected area network for the conservation of Mediterranean heritage [17]. Of growing importance is the Pan European Ecological Network, which includes the European Union Natura 2000 network [23], [24] and the Emerald network of the Bern Convention [25]. Other Eurocentric policies include the Marine Strategy Framework Directive (MSFD) [26], which requires the European States of the Mediterranean to prepare national strategies to manage their seas to achieve or maintain good environmental status by 2020 [27]. In contrast with these conservation and management initiatives and conventions, the Ramsar Convention includes member states throughout the Mediterranean Basin and focuses on a single threatened habitat, coastal wetlands ( Text S1 in *Supporting Information*). These mandates and initiatives require that areas and actions are prioritized to ensure that conservation and management efforts will produce biological and socioeconomic long-term benefits.

These goals could be achieved through systematic conservation planning: the process of locating, implementing and maintaining areas that are managed to promote the persistence of biodiversity and other natural values [28], [29]. In practice, conservation planning has often not been systematic [30]. *Ad hoc* conservation has resulted in conservation and management areas that do not equitably represent regional biodiversity, with boundaries and management regimes that are often determined based on political or economic constraints [14]. In some cases areas have been selected based on their low economic significance rather than consideration for high levels of biodiversity or unique values [31]. Such an opportunistic approach, and the absence of coordinating efforts between relevant parties, has led to inefficient conservation [32]. This is of particular concern considering the very limited resources available for a discipline addressing crises [18], [33].

In the Mediterranean Sea, systematic approaches to conservation prioritization have only been applied at local level, utilizing conservation planning tools such as Marxan e.g., [34], [35], [36], [37]. However, in recent years at least 12 new different regional-scale plans for conservation priority areas have been proposed, in addition to 6 existing ones (see Table 1, Text S1). These plans focus on both multiple and single taxa, and apply a range of criteria and conservation planning approaches and tools. This diversity in perspectives and approaches adopted by different groups responding to the challenges of establishing large-scale conservation plans for the Mediterranean encourages open debate, yet may also point to inefficient conservation planning [32]. Multiple priority setting exercises may reflect different conservation objectives, alternative uses of the available information and varying data quality e.g., [38]. Moreover, all these initiatives involve identifying independent programs to gather data, undertake analyses, and publish and advertise products, creating policy confusion. A similar situation faced global terrestrial conservation at the beginning of the millennium [39], [40]. These efforts may send different or conflicting messages to decision makers, civil society organizations, donors and the public [41], severely partitioning investment of resources and effort. Without careful consideration, the benefits offered by multiple diverse perspectives may be outweighed by the absence of scientific and conservation consensus in setting objectives. Even with these initiatives in place, to date, major areas and priority habitats of the Mediterranean have been overlooked for conservation or MPA designation and marine management [14].

**Table 1:** Main characteristics of the six existing conservation plans in the Mediterranean Sea (additional information in SOM).

Name of Initiative	Type of Organisation	Motivation	Approach^1^	Criteria	Planning Tool	Reference	Map viewable in Figure	% included (of tot Med Sea area)
National initiatives/ Present MPAs	Governmental; aggregation of national initiatives	Legally binding	mainly biodiversity driven; in some indirect socioeconomic and threat considerations; most have been selected opportunistically	various criteria	Mainly expert judgement	Abdulla et al. 2008, Abdulla et al. 2009 (first MPA was established in 1963)	1. a	3.8%
EU/Nationally designated areas (CDDA)	Governmental; aggregation of EU member-states initiatives	Legally binding	mainly biodiversity driven	**Biodiversity**: Designation types used with the intention to protect species, habitats and landscapes; statutes under sectorial, legislative and administrative acts providing adequate protection relevant to species and habitat conservation; private statute providing durable protection for species and habitats	Expert judgement	http://www.eea.europa.eu/data-and-maps/data/nationally-designated-areas-national-cdda-3 (inventory started in 1985)	1. b	1.3%
UNESCO/Marine World Heritage Sites	Inter-governmental Convention	Legally binding	Cultural and Biodiversity driven	**Biodiversity**: important biological and ecological processes for the evolution of species, important habitats for high biodiversity and for threatened species. **Geological**: significant geological processes and geomorphic features. **Cultural and aesthetic uniqueness**	Expert judgement	http://whc.unesco.org/en/list (first marine world heritage site listed in 1983)	1. c	N/A
Barcelona Convention SPA-BD protocol/Specially protected areas of Mediterranean Importance (SPAMIs)	Inter-governmental treaty	Legally binding	Biodiversity driven; consideration of scientific, aesthetic, cultural or educational importance	**Biodiversity (species distribution and habitats**): 5 main criteria: 1. Uniqueness; 2. Natural representativeness; 3. Diversity; 4. Naturalness; 5. Presence of habitats that are critical to endangered, threatened or endemic species; and **Cultural representativeness**	Expert judgement, political decision (nomination of sites by the party/parties concerned)	SPA/BD 1995	1. c	N/A
EU/Natura 2000 network	Inter-governmental body (EU)	Legally binding	Biodiversity driven	**Biodiversity (species distribution and habitats**): 9 broad habitat types and 10 priority species (relevant for the Mediterranean); criteria: representativeness, % coverage, size, degree of conservation, degree of isolation, evaluation of the overall value of the site for priority species and habitats	Expert judgement	Habitats Directive (EC, 1992); Birds Directive (EC, 2009); Process initiated in 1996 – on-going expansion of the network	1. d	1.3%
Ramsar, Convention on Wetlands	Inter-governmental treaty	Legally binding	Biodiversity driven	**Biodiversity (species distribution and habitats**): 9 criteria for the importance of the included habitats, bird and fish populations, endangered species and communities, and their role as spawning/nursery areas, feeding grounds, or migration paths (see details in Suppl. 1)	Expert judgement, quantitative data and analysis	Ramsar Convention Secretariat, 2010 (strategic framework and guidelines first adopted in 1999)	1. e	0.1%

1 Biodiversity driven: The priority areas were selected considering biophysical data only; Threats: The priority areas were selected after consideration of biophysical data and threats to habitats/species/ecosystems; Socioeconomic considerations: The priority areas were selected after consideration biophysical and socio-economic data and were located in places were conservation goals where achieved with minimum socio-economic cost.

There are at least three different strategies for addressing the disparate spatial conservation prioritization initiatives and proposals. First, one could initiate an entirely new effort. Given the abundant existing work, this would not be a productive approach. Second, one could select a proposal among the existing ones (Table 2) that should be brought forward toward implementation. This is expedient and there are strong arguments for taking this path. In particular, the only formal process for this region for the identification of priority conservation areas was led by the United Nations Environment Program's Mediterranean Action Plan in 2009 (hereafter “UNEP MAP”) in cooperation with the European Commission. This led to the identification of a set of large Ecologically or Biologically Significant Areas (EBSAs) distributed throughout the basin (Table 2) [42]. The EBSAs process has been endorsed by all the contracting parties to the Barcelona Convention (21 Mediterranean countries and the European Union). This formal commitment renders this proposal the most likely to guide conservation planning in the Mediterranean (Portman et al., unpublished data). However, the expert-judgement approach used in the selection of EBSAs, and a focus on offshore and pelagic habitats, may have led to underrepresentation of important areas. Thus, it is critical that all available sources of information are used to determine what conservation features (i.e. habitats and species) were ‘left out’, or under-represented, in the process. Moreover, EBSAs cover a large portion of the Mediterranean (36.5%; Table 2); hence it may be necessary to select priority sites for protection and management within these large areas.

**Table 2:** Main characteristics of the twelve proposed conservation plans in the Mediterranean Sea (additional information in SOM).

Name of Initiative	Type of Organisation	Motivation	Approach1	Criteria	Planning Tool	Reference	Map viewable in Figure	% included (of tot Med Sea Area)
WWF MedPO/13 key Mediterranean marine areas in need of protection	NGO	unsolicited	Biodiversity driven, Threats	**Biodiversity (species distribution and habitats**): monk seal, marine turtles, species of whales and dolphins, seagrass P. ocenica and sea-bed heterogeneity to estimate fish species diversity **Threats**: Pollution, urbanization (large harbours and coastal cities)	GIS, Gap analysis	awsassets.panda.org/downloads/background.doc, 1998	2. a	24%
GFCM/Fisheries restricted areas	Scientific committee for fisheries (MED)	requested scientific advice	Biodiversity driven (emphasis on the conservation of fisheries resources)	**Biodiversity (habitats**): deep sea sensitive habitats; spawning aggregations	Expert judgement	Recommendations GFCM/2005/1, GFCM/2006/3, GFCM/33/2009/1	2. b	58.5% (FRAs: 0.7%; trawling ban >1000m: 57.8%)
Greenpeace/Marine reserves for the Mediterranean Sea	NGO	unsolicited	Biodiversity driven	**Biodiversity (species distribution and habitats**): Distribution of species (including whales, dolphins, seals and fish); important areas for marine species (such as spawning grounds, nursery areas and nesting beaches); important habitats (such as seagrass meadows); **Geological**: seamounts **Previous initiatives**: SPAMI and Natura 2000 sites	Expert judgement, GIS, quantitative analysis	http://www.greenpeace.org/france/PageFiles/266559/marine-reserves-med.pdf, 2006	2. c	54.5%
ACCOBAMS/Existing and proposed MPAs for whales and dolphins in the Mediterranean and the Black Seas	Regional Scientific Agreement	unsolicited	Biodiversity driven, qualitative consideration of threats	**Biodiversity (species distribution and habitats**): Critical habitats for Cetaceans (e.g. feeding and breeding areas); **Oceanographic**: upwelling areas; Geological: canyons	Expert judgement	http://www.accobams.org/images/stories/Guidelines/mpas%20guidelines.pdf, 2007	2. d	13.5%
Vulnerable habitats	Scientific cooperation	unsolicited	Biodiversity driven (emphasis on the conservation of fisheries resources)	**Biodiversity (habitats**): Protection of Essential Fish Habitats (spawning grounds, nursery areas, migration routes) and Sensitive Habitats (deep-water corals and cold hydrocarbon seeps). **Geological**: seamounts, canyons	Expert judgement	De Juan and Lleonart 2010	2. e	11.3% (9.6% pelagic; 1.7% demersal)
UNEP MAP RAC-SPA/Important sea birds areas	Inter-governmental body	Requested scientific advice	Biodiversity driven	**Biodiversity (species distribution**): seabird species richness and vulnerability **Oceanographic**: bathymetry, sea surface temperature, sea productivity (Chlo-a) **Geological**: seamount proximity, shoreline	Expert judgement, quantitative data and GIS	UNEP MAP-RAC/SPA, 2010. Carboneras and Requena, 2010.	2. f	40.9%
UNEP MAP RAC-SPA and EU/Ecologically or Biologically Significant Areas (EBSAs)	Inter-governmental body	requested scientific advice	Biodiversity driven	**Biodiversity (species distribution and habitats**): Marine birds, cetaceans, monk seals, marine turtles, sharks, bluefin tuna, deep-sea coral reefs, coralligenous facies, white coral communities, commercial pelagic fishes (spawning and nursery areas); **Oceanographic**: bathymetry, primary productivity, SST; **Geological**: seamounts, canyons, mud volcanoes, hydrothermal vents; **Previous initiatives**: Fisheries Restricted Areas (GFCM); UNEP MAP RAC-SPA/Important sea birds areas	Expert judgement, GIS	UNEP-MAP-RAC/SPA, 2010. UNEP-MAP-RAC/SPA, 2012	2. g	36.5%
CIESM/Mediterranean Marine Peace Parks	Mediterranean Scientific Commission	unsolicited	Biodiversity driven & intergovernmental collaboration for peace	**Biodiversity (species distribution and habitats**): endemic & threatened biota (such as unique deep-sea communities, white coral beds, monk seals, rare endemic species, fin whales, spawning grounds of bluefin tuna); **Oceanographic**: surface sites of deep water formation); **Geological**: deep sea canyons, mud volcanoes, seamounts, hypersaline craters	Expert judgement	CIESM, 2011	2. h	target: >10%; no specific boundaries defined
Oceana/MedNet: MPA network proposal for the Mediterranean Sea	NGO	unsolicited	Biodiversity driven, qualitative consideration of threats, administrative and legal considerations, existing proposals	**Biological (species distribution and habitats**): Key species; CBD criteria. **Oceanographic**: Connection by currents, gyres; Fronts **Geological**: escarpments, seamounts, canyons, trenches, etc.. **Threats**: Illegal, Unregulated and Unreported Fishing; Potential oil and gas prospecting; Pollution; Maritime traffic; By-catch. **Administrative**: Affected by waters of national jurisdiction; Jurisdictional conflicts. **Previous initiatives**: ACCOBAMS; EBSAs; De Juan & Lleonart, 2010; GFCM (FRAs); Greenpeace	GIS, quantitative data and analysis	Oceana 2011	2. i	8.2%
Cumulative Impact Map	Scientific consortium	unsolicited	Biodiversity and Threats	**Biodiversity (habitats**): 19 habitat types (e.g., rocky reef, seagrass beds, epipelagic areas, deep soft sediments).**Threats**: 18 spatial datasets of human activities and stressors (including fishing, coastal population density, hypoxia, invasive species, pollutants and nutrients, ocean acidification, oil accidents, oil rigs, Sea Surface Temperature change, shipping, urbanization trends, UV change)	GIS, quantitative data and analysis	Micheli et al. 2011 http://globalmarine.nceas.ucsb.edu/mediterranean/	2. j	5.6%
Fish Biodiversity	Scientific consortium	unsolicited	Biodiversity driven and Threats	**Biodiversity (fish**): total species richness, endemic species richness, IUCN threatened species richness, phylogenetic diversity, functional diversity. **Threat**: fishing pressure	GIS, quantitative data and analysis	Mouillot et al. 2011	2. k	11%
Conservation Concern Areas	Scientific consortium	unsolicited	Biodiversity and Threats	**Biodiversity (species distribution**): marine mammals, turtles, seabirds, fishes and commercial or well-documented invertebrates; **Threats**: coastal-based impacts, trawling and dredging disturbance, ocean-based pollution, exploitation of marine resources by fisheries, maritime activities and impacts of climate change.	GIS, quantitative data and analysis	Coll et al. 2012	2. l	13.7%

The third approach is to integrate the different regional-scale conservation plans proposed thus far for the Mediterranean Sea. By integrating these different efforts, we identify priority conservation areas that represent a consensus. Building upon this consensus we provide a framework to guide future progress in prioritizing actions within these key areas. Here, we (i) review the existing and proposed spatially-based conservation plans for the Mediterranean Basin, (ii) synthesize and integrate the different plans to determine current consensus regarding top priority areas, and (iii) discuss a general framework, based on the principles of systematic conservation planning, for identifying priority areas and actions for future application to the Mediterranean region.

## Methods

### Review of existing and proposed regional conservation plans

Six existing and twelve proposed regional initiatives for the conservation of the Mediterranean Sea were identified after a thorough investigation of the peer-reviewed and grey literature. For each initiative, documents supporting the existing plans or proposals were reviewed and the main features of each initiative were extracted and summarized in Tables 1 (existing) and 2 (proposed). More detailed information is provided in Text S1.

The following characteristics of each initiative were recorded: organization promoting the initiative; type of organization; motivation (solicited, unsolicited, legally binding); approach (biodiversity driven: the priority areas were selected considering ecological features; threats: prioritization by assessing threats to habitats/species/ecosystems; socioeconomic considerations: selection after consideration of biophysical and socio-economic data and prioritization of places where conservation goals are achieved with minimum socio-economic cost); criteria (what biodiversity, oceanographic, geological, threat etc. data were included); methods and planning tool (e.g. expert judgment, qualitative analysis, geographic information systems [GIS]); main scientific reference of the initiative; and the extent of existing or proposed protection (the percentage of the Mediterranean selected as a priority conservation area, Tables 1–2).

### Map development

Spatial data were gathered from various sources for the initiatives included in our review, as listed in Tables 1 and 2. For most of the initiatives, we were given access to the original GIS layers (shape files) either directly from the authors or from relevant websites (Present MPAs, EU CDDA, SPAMI, Natura2000, Ramsar sites, ACCOBAMS, EBSAs, Oceana) [6], [43], [44] (see Tables 1–2). For the remaining initiatives, we georeferenced raster maps and then digitized the priority areas proposed by the initiative. All layers were projected to the Lambert Azimuthal equal area projection to allow for the calculation of the area covered by each of the initiatives. Subsequently, we calculated the number of times a 10 km^2^ grid cell was included in existing plans, and the number of times a 10 km^2^ grid cell was included in proposed initiatives.

We calculated the correspondence between the 12 regional proposals (Table 2) to evaluate the similarity between them. We used confusion matrices [45] to calculate the overall accuracy and overall correspondence between all possible pairs of the different proposals. A confusion matrix is a quantitative comparison between classes (in our case, binary maps of priority areas) that were derived by different algorithms (in our case, the proposals). Thus, in our analysis each confusion matrix had two columns and two rows, for the four possible combinations of all the pairs of two binary maps. As in most cases priority areas cover a small proportion of the total Mediterranean Sea, overall accuracy estimates are inflated. Therefore we calculated the Kappa Index of Agreement [46], which expresses the proportion of correct classification above the expected proportion corrected due to chance.

## Results

### Existing and proposed Mediterranean marine conservation areas range widely in extent and location

**Figure 1:**
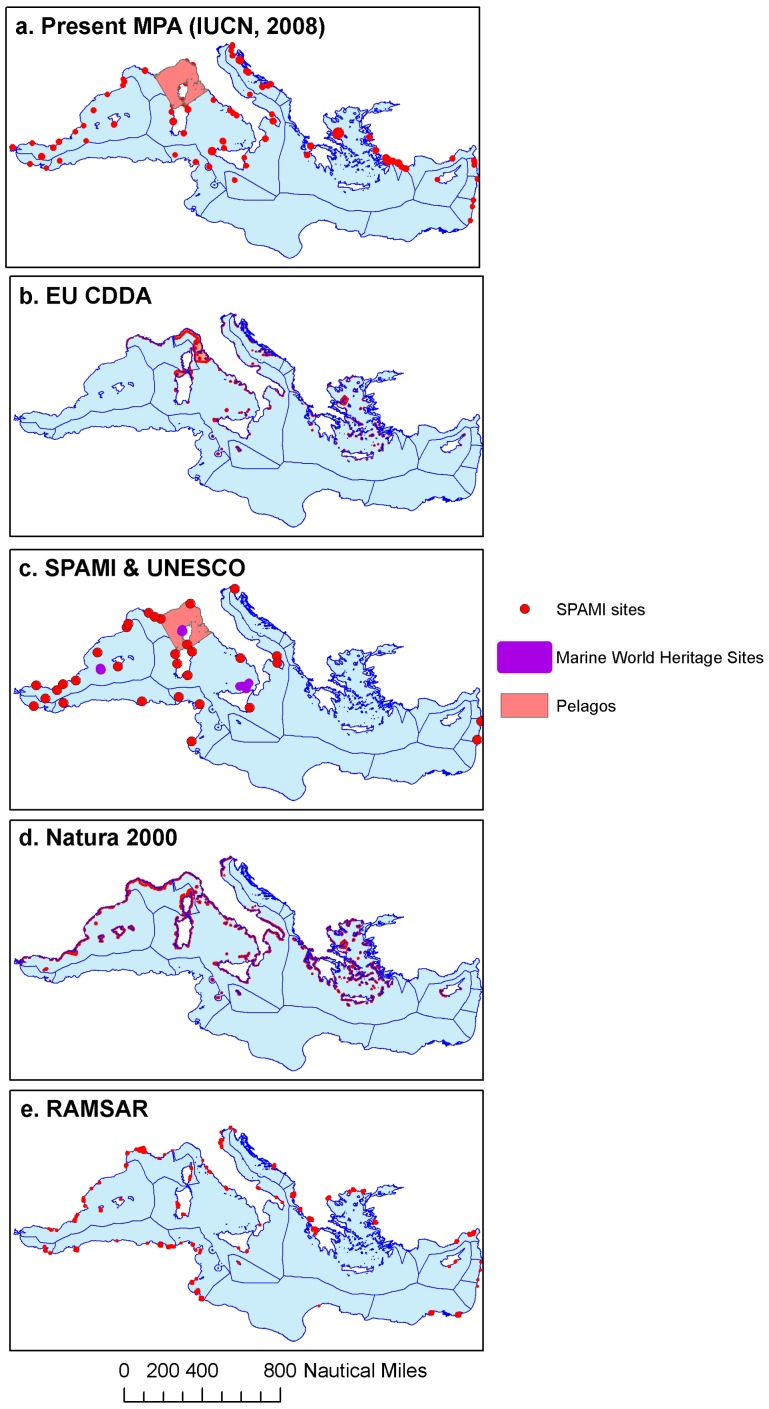
Existing marine management and conservation areas in the Mediterranean Sea (see **Table** **1A** for descriptions).

**Figure 2:**
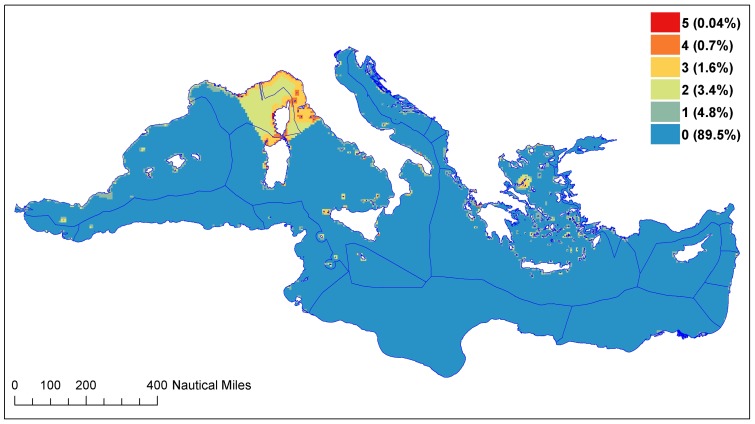
Frequency of inclusion by existing marine management and conservation areas. The number of schemes including a particular area and the total % included are reported in the legend.

Existing conservation in the form of MPAs that have already been designated (Table 1 and Text S1) is almost exclusively coastal (Fig. 1). The only exception is the Pelagos Sanctuary for Mediterranean Marine Mammals, which includes a large area of offshore waters. In total, existing MPAs include between 0.1% – 3.8% of the Mediterranean, depending on the initiative considered and the MPA definition used (Table 1). Additionally, the majority of MPAs are located along the western and northern shores, with the exception of the Ramsar sites (Fig. 1). Ramsar sites are designated for the conservation of a single habitat type, wetlands, and only to a maximum depth of 6 m (Table 1, Text S1). This coastal focus and the broad participation of non-EU member states in the Ramsar Convention likely underlie the greater representation of North African shores. However, even when all existing marine conservation areas are considered simultaneously, the under-representation of the eastern and southern portions of the basin and of offshore waters is apparent (Fig. 2).

**Figure 3:**
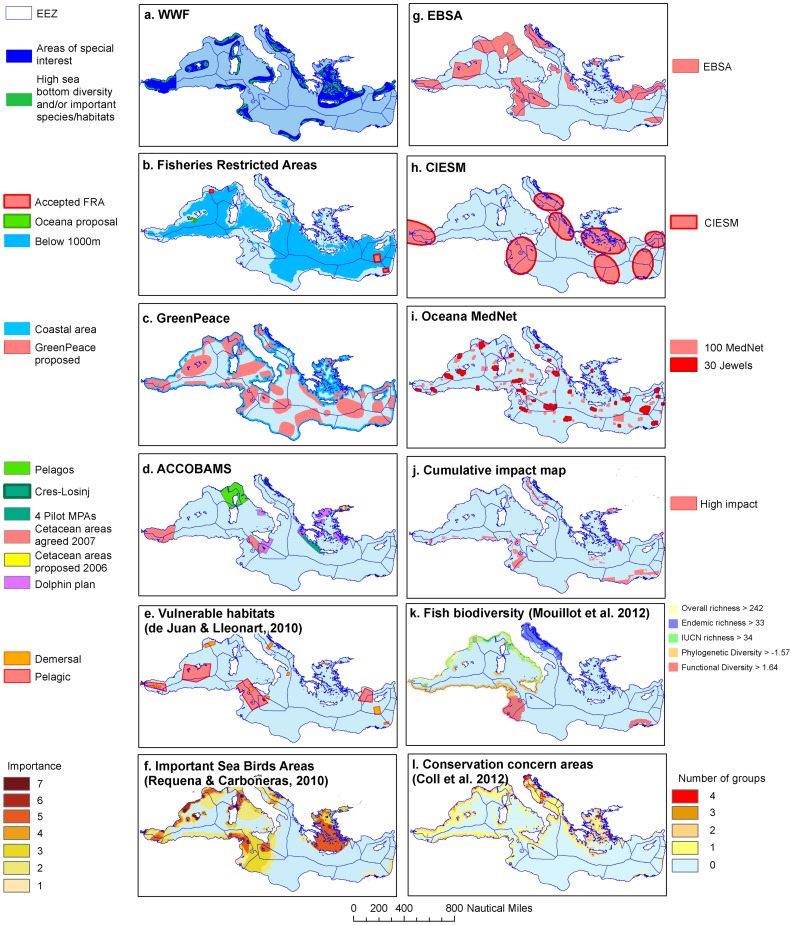
Proposed conservation priority areas in the Mediterranean Sea (see **Table** **1B** for descriptions).

The bias towards the western and northern region of the basin is also clear in some of the proposed conservation plans (Fig. 3, Table 2, Text S1) including the proposals by ACCOBAMS (Fig. 3D), the Important Sea Bird Areas (Fig. 3F), and the Areas of Conservation Concern (Fig. 3L). However, the majority of proposals identify conservation areas that are more representative of different Mediterranean ecoregions than is currently conserved or managed. The areas proposed by Greenpeace (Fig. 3C), Oceana (Fig. 3I), the Convention on Biological Diversity EBSAs (Fig. 3G), and the CIESM Marine Peace Parks (Fig. 3H) reflect a broader consideration of the region. In addition, most of the proposed conservation areas encompass both coastal and offshore (pelagic and/or demersal) ecosystems, which starkly contrasts with the existing MPAs (Fig. 1).

Different initiatives vary in the total extent of proposed conservation areas (Table 2). Most initiatives propose a total extent of protection or management in the range of ∼7–14% of the Mediterranean. Notable exceptions are the proposals for Important Sea Bird Areas (Fig. 3F) and the EBSAs (Fig. 3G), which include 40.1% and 36.5% of the total surface area of the Mediterranean, respectively (Table 2). The lowest percent coverage is for the Fisheries Restricted Areas (FRAs of the General Fisheries Council of the Mediterranean, GFCM) which proposes an area of 0.7%, excluding the trawling ban at depths >1000 m (Fig. 3B, Table 2). Inclusion of marine areas with depths >1000 m would increase the proposed FRAs to 58.5% of the Mediterranean. The Greenpeace initiative proposes a similar overall percentage of coverage, 54.5% (Fig. 3C), which is the largest coverage of any of the proposals. These differences partly stem from the legal foundation of some of the initiatives (Table 2) that restrict the geographic scope of the conservation effort (e.g. to the territorial waters of EU countries) or the management objective (e.g., the protection of birds or mammals). In contrast, there are no such restrictions in the case of unsolicited initiatives (Table 2), including those by Greenpeace, WWF, Oceana, and CIESM.

### Different criteria and data are used for selecting priority conservation areas in the Mediterranean

The wide variation in the proposed priority conservation areas (Fig. 3) can be explained by differences in the objectives, criteria and data used by the different initiatives. Both for existing (Table 1, Fig. 1) and proposed (Table 2, Fig. 3) conservation areas, the considerations and criteria used for identifying priorities are primarily driven and informed by biodiversity conservation goals and biophysical criteria. Conservation goals are most commonly developed for the protection of species, habitats and seascapes, with some initiatives focusing more narrowly on specific threatened and charismatic taxa (e.g., cetaceans, sea birds, large pelagic fish, deep sea corals; Table 2 and Text S1). Some initiatives have additional goals of maintaining ecosystem services, promoting the sustainable use of natural resources, and endorsing cooperation among countries (CIESM, GFCM FRAs; Text S1).

**Figure 4:**
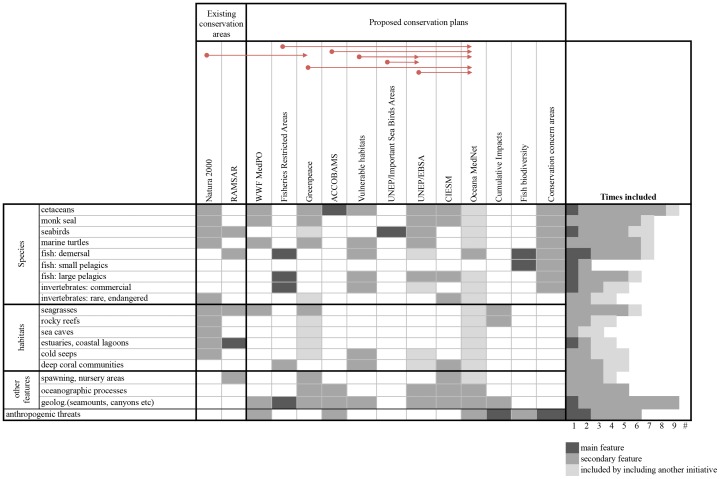
Main features and considerations for the selection of existing and proposed conservation areas. The number of times a specific feature was considered in different initiatives is reported. Some proposals incorporated existing initiatives and plans: these are indicated by the light grey boxes and the red arrows. Among the existing conservation areas, SPAMIs, EU CDDA, and existing MPAs were not included because they are aggregations of protected areas based on different criteria.

Among the criteria used for area selection, species distribution, particularly for marine mammals, seabirds, sea turtles and demersal and large pelagic fishes was considered most frequently in the different initiatives (Fig. 4). Oceanographic and geologic features, such as upwelling processes and the distribution of seamounts and deep canyons were also widely considered (Fig. 4). In contrast, few initiatives included data on small pelagic fishes or invertebrates. The intensity and distribution of anthropogenic threats was considered, either qualitatively or quantitatively, by 6 of the 12 proposals (Fig. 4), with only 2 quantitative assessments [6], [43]. It is important to note that several areas of overlap exist as some proposals take into account previous initiatives in their prioritization scheme (light grey cells in Fig. 4). This was particularly evident in the Oceana MedNet, and, to a lesser extent, in the EBSAs and the Greenpeace proposal (Fig. 4).

Some initiatives were largely based on expert judgement due to a lack of quantitative data for parts of the region, especially the southern and eastern Mediterranean Sea (Tables 1 and 2). Expert judgement and spatial data on biological, oceanographic and geological features were mapped in GIS layers. The areas where such layers overlapped were identified as priority areas for conservation (Tables 1 and 2, Text S1). Two initiatives, the Cumulative Impact Map (Fig. 3J) and Areas of Conservation Concern (Fig. 3L), used data layers of the spatial distribution of threats, in combination with habitats [43] (Fig. 3J) or species diversity [6] (Fig. 3L) to identify priority conservation areas. Some existing MPAs, particularly those in the SPAMI list, were established based on biophysical, cultural, social or economic considerations addressing human values and feasibility of protection, although the inclusion of these criteria varied greatly among locations (Table 1, Text S1). In contrast, none of the proposed conservation plans explicitly included feasibility or socioeconomic data as criteria for identification of priority areas (Table 2 and Text S1). The CIESM proposal is the only one to have the explicit political goal of fostering intergovernmental collaboration (Table 2, Fig. 3H).

### Consensus exists among the different initiatives

**Figure 5:**
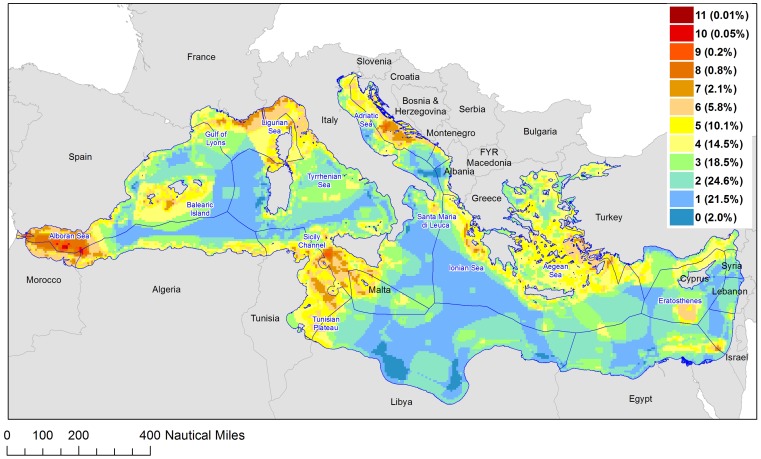
Frequency of inclusion by proposed conservation plans. The number of schemes including a particular area and the total % included are reported in the legend.

Despite wide variation in the size and location of conservation areas proposed by the different initiatives (Fig. 3), their overlap reveals clear consensus for some areas (Fig. 5). These spatial overlaps can be considered as consensus areas and therefore top priorities as their selection was robust to variation in the objectives and criteria guiding the different proposals.

Areas within the Alboran Sea were selected by all of the initiatives considered (Fig. 5). Thus, these areas represent the strongest consensus as a conservation priority. Other areas that were selected by a majority of the initiatives (6 or more), and are therefore considered as representing strong consensus, include areas within the Sicily Channel and the Tunisian Plateau, areas around the Balearic Islands, the Gulf of Lyons, areas in the Ligurian and central Tyrrhenian Sea, the central and northern Adriatic Sea, the inner Ionian Sea, the eastern Aegean Sea, waters off Israel and Egypt, and the Eratosthenes and Santa Maria di Leuca seamounts (Fig. 5). These areas represent the current strongest consensus on conservation priorities. Taken together, these top 10 priority areas encompass approx. 10% of the Mediterranean Sea. Areas selected by at least 5 proposals encompass an additional 10% of the basin, largely within the same regions listed above (Fig. 5).

**Figure 6:**
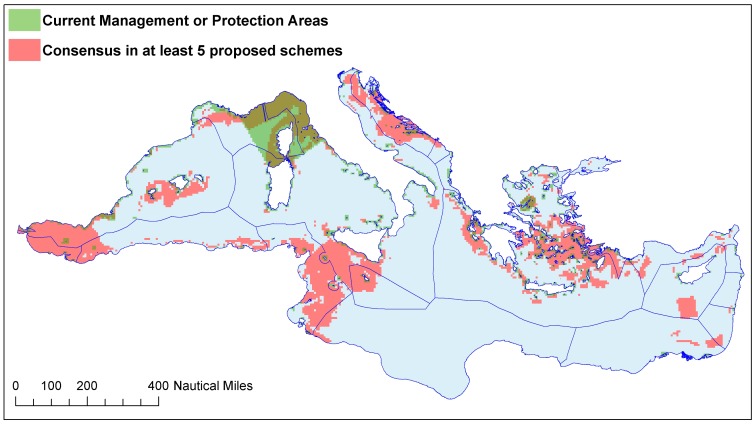
Overlap between existing conservation areas (light green) and proposed conservation priority areas (pink). Conservation priority areas were those selected by at least five initiatives. Overlap between existing and proposed areas is indicated by the dark green color.

Comparison of the overlap maps of existing (Fig. 2) and proposed (Fig. 5) conservation plans helps identify critical conservation gaps (Fig. 6). In particular, this comparison highlights that large areas proposed by multiple initiatives are currently not included in any of the existing conservation schemes (pink areas, Fig. 6).

Based on the confusion matrices (Table 3), the FRAs initiative (GFCM) was the least similar to other existing or proposed plans, with an average overall low accuracy of 38% (average kappa index of −17%). This is probably due to the definition of all areas below 1,000 meters as important for conservation. The ACCOBAMS initiative was the most similar to other initiatives with an average overall accuracy of 72% (average kappa index of 11%), followed by the EBSA initiative (average kappa index of 12%; Table 3). The high similarity of the ACCOBAMS proposal to others is explained by the fact that most initiatives included the distribution of cetaceans in their criteria. The two most similar proposed plans were the EBSAs and the Vulnerable Habitats [47], with an overall accuracy of 85% (kappa index of 46%). This is at least partly due to the incorporation of the Vulnerable Habitats [47] results in the EBSAs selection process (Fig. 4).

**Table 3:** Correspondence between the different proposed conservation plans. Values above the diagonal (marked by empty cells) are the overall correspondence between pairs of proposals.

	WWF	Fisheries	GreenPeace	ACCOBAMS	EBSA	Vulnerable habitats	Sea Birds	CIESM	OCEANA	Cumulative impacts	Conservatio Concern	Fish biodiversity
WWF		35%	59%	74%	69%	70%	64%	69%	70%	71%	76%	72%
Fisheries	−23%		37%	40%	44%	44%	25%	38%	48%	42%	35%	32%
GreenPeace	22%	−28%		56%	50%	47%	59%	50%	47%	46%	56%	53%
ACCOBAMS	20%	−12%	18%		78%	81%	68%	68%	81%	82%	81%	78%
EBSA	15%	−6%	4%	27%		85%	64%	63%	75%	77%	70%	73%
Vuln. habitats	1%	−3%	2%	11%	46%		61%	69%	86%	87%	78%	80%
Sea Birds	21%	−47%	20%	26%	20%	9%		59%	57%	60%	68%	67%
CIESM	22%	−20%	4%	8%	5%	8%	12%		68%	71%	67%	68%
OCEANA	−3%	4%	1%	−2%	5%	11%	−1%	1%		88%	80%	80%
Cum. impacts	0%	−6%	1%	1%	9%	7%	5%	9%	0%		82%	86%
Cons. concern	25%	−21%	17%	18%	0%	−7%	26%	7%	−6%	1%		82%
Fish biodiv.	12%	−26%	12%	4%	9%	2%	23%	7%	−5%	16%	21%	
**Av. overall accuracy**	**66%**	**38%**	**51%**	**72%**	**68%**	**72%**	**59%**	**63%**	**71%**	**72%**	**70%**	**70%**
**Av. kappa index of agreement**	**10%**	**−17%**	**6%**	**11%**	**12%**	**8%**	**10%**	**6%**	**1%**	**4%**	**7%**	**7%**

Values below the diagonal are the kappa index of agreement (see *Methods*).

## Discussion

This study provides a review of the multiple existing and proposed conservation achievements and plans within the Mediterranean. Importantly, it highlights the consensus regarding top priority areas selected through these different planning processes. We found that a majority of plans share similar goals and criteria despite differing in the type of data used to describe biophysical features (Tables 1 and 2, Fig. 4). Data ranged from the distribution of specific taxa and habitat maps to measures of diversity, such as species or functional diversity. The initiatives also differed in the approaches used, ranging from qualitative expert surveys to the use of multiple datasets that are integrated into spatial models. Spatial distribution of threats to species, habitats, and ecosystems were considered only in a small subset of initiatives (Tables 1and 2, Text S1). None of the initiatives explicitly incorporated socioeconomic data or goals. Finally, there was no consideration of the feasibility of implementing conservation in the areas selected, or the conditions that may provide opportunities for progress and recovery in the short term.

A large fraction of the Mediterranean (40.2%) was selected by at least one proposal. This figure highlights the strong influence of the criteria considered, and the availability and quality of the data conducive to the selection of a range of different priority conservation areas. The differences in the criteria and approaches used in the proposed plans, as well as data gaps, may also explain the under-representation of some Mediterranean regions, particularly the southern and eastern portions of the basin. For example, initiatives that considered geological features such as seamounts tend to include more areas in the south-eastern Mediterranean Sea (Figs. 3 and 4). Biodiversity data are scarce for the south and eastern regions of the Mediterranean, therefore initiatives that relied primarily or exclusively on these data tended to under select these areas e.g., [3], [6]. Finally, a lack of social, economic, cultural and political criteria also underlies the selection of unrealistically large areas and regions where international collaboration towards transboundary conservation is unlikely for political reasons. It will be critical to include these criteria and considerations in future analyses.

### Current gaps and recommended approaches

Our approach to addressing data gaps and variation among the criteria adopted by the initiatives was to leverage the complementarity of different approaches to identify outcomes that were robust to this variation. However, several issues remain. Systematic conservation prioritization schemes should implicitly take into account the spatial variability of anthropogenic uses and the associated cost of excluding uses for conservation needs [48], [49], [50], [51]. The establishment of MPAs or other management measures in priority conservation areas may restrict economic activities, particularly extractive industries. In human-dominated environments, like the Mediterranean Sea, such considerations cannot be disregarded. For instance, the Eratosthenes seamount is among the priority areas for conservation we identified. However, none of the initiatives accounted for the economic importance of this area for Cyprus due to the natural gas and oil deposits found here [52], [53]. Indeed, the Eratosthenes seamount was originally included in the EBSAs initiative but was later removed due to the objection of Cyprus [42]. Steps for including costs in regional conservation prioritization, within heavily exploited regions, could use spatial optimisation tools (e.g. Marxan) to achieve targets with minimum cost.

The Mediterranean Sea is home to ∼100 marine biotopes [54]. Many marine biotopes (especially offshore, pelagic and deep seabed habitats) are underrepresented or absent in existing conservation areas [14]. Fine-scale habitat mapping is largely lacking especially in data poor regions such as the southern and eastern Mediterranean. These gaps in knowledge are reflected in newly proposed conservation areas (Table 2, Fig. 4) where, apart from seagrasses and some deep benthic habitats, the bulk of marine biotopes have been ignored during prioritization processes. As many Mediterranean habitats are vulnerable to a number of human pressures and have been facing substantial deterioration [55], further effort and funds should be invested for ecological mapping, especially in data-poor regions.

Similarly, our knowledge of the population status and distribution of many species, especially invertebrates, is clearly insufficient. From the 35 invertebrate species included in Annex II of the Protocol for Specially Protected Areas and Biological Diversity in the Mediterranean of the Barcelona Convention, only one species (the gastropod *Gibbula nivosa* – one of the Annex II species of the Habitats Directive) was considered in one of the initiatives (NATURA 2000). All other species were not specifically considered in the prioritization process. This is indicative of a lack of knowledge of the spatial distribution and habitat requirements of threatened species. A promising approach in this regard is represented by Species Distribution Models (SDMs) [56]. These are numerical tools that combine observations of species occurrence with environmental variables to predict the probability of the presence of a species even for areas that have not been sampled. SDMs have been widely used in the terrestrial environment for a number of theoretical and applied questions e.g. [57], but applications in the marine realm remain relatively scarce [58]. Yet, SDMs can potentially help in filling the gap of knowledge on species distribution/presence for poorly known areas, as it has been clearly demonstrated for terrestrial organisms e.g. [59].

It is also important to perform any conservation planning exercise while considering the entire set of different bioregions and/or ecoregions that characterize the Mediterranean basin e.g., [11]. Explicitly considering these regions in a conservation plan would help limit the regional bias existing in the available data on species distribution [58], [60], [61] and ecological features [3], thereby ensuring a full consideration of the entire set of ecological and biological features that characterize the region.

However, the absence of high-quality information on habitats and species distribution and status from some regions cannot be an excuse for inaction in the Mediterranean Sea [14]. The rapid degradation of Mediterranean ecosystems e.g. [5], [6], [15], [55] dictates the urgent need for setting priorities for regional conservation planning and for taking management measures that could be modified later with the improvement of our knowledge. Many countries in the Mediterranean cannot afford to implement comprehensive research on all marine habitats and species within their national jurisdiction. Under these circumstances, a different approach may be necessary, whereby the information required for the designation of MPAs or marine management measures arises through the integration of available information with rigorous quantitative research in a few representative sites, combined with comprehensive surveys of traditional knowledge [62].

### Consensus among the different initiatives: identifying the top conservation priorities for the Mediterranean Sea

Our results highlight consensus among the initiatives reviewed, which allows for the identification of areas where actions may be prioritized. The review and integration of the 12 Mediterranean conservation proposals highlights 10 priority areas, covering ∼10% of the Mediterranean Sea. In addition, a further 10% of the Mediterranean Sea was selected (around these core areas) by at least five of the initiatives (Fig. 5), resulting in a total ∼20% of the region that represents full or partial consensus. These areas provide a proposal that is robust to the differences in the methodology and data guiding the different conservation initiatives. The implementation of conservation actions within the areas of consensus would greatly enhance the extent and representativeness of conservation areas in the Mediterranean Sea (Fig. 6).

Areas within the Alboran Sea were selected by all of the initiatives considered. The Alboran Sea encapsulates the fundamental problem of balancing human use with nature conservation. It hosts important natural habitats and is the only entrance into the Mediterranean Sea from the Atlantic Ocean, making it important for both migratory species and shipping. Activities affecting this marine region include demersal fishing and commercial shipping (Micheli et al. 2011). Fundamental for this region is to spatially separate its multiple uses, whilst conserving a representative sample of the ecosystems. Similarly, multiple fisheries affect all other areas that were selected by more than half of the initiatives, therefore considered as representing a strong consensus for protection. In addition, commercial shipping is an important pressure on the ecosystems of the Sicily channel, and land based activities leading to coastal pollution and hypoxia affect areas in the Adriatic and Tyrrhenian Seas (Micheli et al. 2011). Similarly to the Alboran Sea, specific conservation actions are also needed to address the challenges that face these areas.

Although these top priorities for conservation in the Mediterranean Sea should be examined under the spectrum of feasibility, socio-economic values, and opportunity costs, they represent a robust and current consensus that can inform decision makers, NGOs, and donors regarding where effort and resources should be most urgently directed. This result has the potential to contribute to the commitment by the Convention of Biological Diversity (CBD) to achieve a significant reduction of the current rate of biodiversity loss, protecting 10–30% of marine habitats by 2020. As discussed above, additional areas should be identified in the south and eastern parts of the Mediterranean using oceanographic data, SDM [57], and traditional knowledge [62] to complement this regional proposal and to meet national commitments to the CBD.

### A roadmap for conservation of the Mediterranean Sea

The selection of priority conservation areas is only one step in strategic conservation planning. In order to move regional conservation plans towards implementation, several additional steps and processes are required. In particular, it is critical that actions are prioritized with the goal of allocating limited resources to effectively minimise or reverse the loss of biodiversity and ecosystem services [28], [38]. Moreover, much like a cake recipe may change slightly according to different tastes, the stages and processes leading to prioritization of conservation actions can differ between or within priority areas. However, there is a need to follow a core sequence of steps to ensure useful and effective conservation.

**Figure 7:**
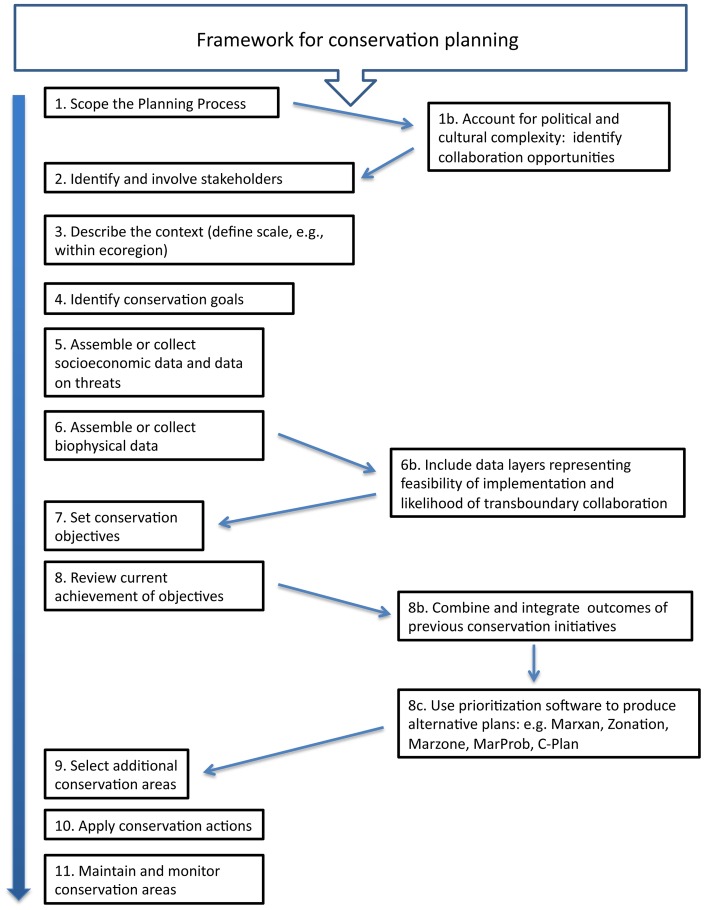
Proposed framework for regional marine conservation planning. The 11 stages of conservation planning presented in Pressey and Bottrill (2009) are on the left, and the additional steps we propose for effective conservation planning within complex marine regions, such as the Mediterranean Sea, are added to the right.

Pressey and Bottrill 2009 [41] propose a framework that includes 11 core steps for systematic conservation planning. Here, we build on this existing general framework to include the complexities that characterise the Mediterranean region (Fig. 7). Based on our analysis and review, we propose 4 additional steps to be explicitly added to those described in Pressey and Bottrill 2009 [41]. Political complexities, and specifically the feasibility of establishing collaborations among stakeholders, regional initiatives, and nations, should be considered both qualitatively during the initial scoping phase (step 1b in Fig. 7), and quantitatively later (step 6b). In politically complex situations, such as in the Mediterranean basin, to ignore these issues is likely to disrupt the entire conservation planning effort. In regions that are the focus of multiple conservation initiatives, we recommend that an additional step is included to synthesize the outcomes of previous conservation plans (step 8b), as has been done in this study. Finally, prioritization software and modelling tools, including Marxan and MarZone, have been developed to produce alternative plans that simultaneously account for conservation targets and constraints identified in the previous steps, and future scenarios of change (including climate, ecological and socioeconomic change). Such tools provide a powerful means of integrating diverse data and considerations to produce priorities (step 8c).

There are several other marine regions facing similar problems related to coordination between multiple conservation strategies [63]. The coral triangle is one such example where multiple organisations and initiatives are in place and consensual agreement would lead to more effective use of financial conservation resources and better governance [64]. Other regions such as the wider Caribbean and the Eastern Tropical Pacific could also benefit from synergy among the multiple ongoing international initiatives.

Like the Mediterranean Sea, complicating these areas are the multiple States that use the regions. In the Mediterranean, the majority of the areas will require cooperation between two or more States. Conservation planning is complex enough in one country, and combining two of more countries in transboundary conservation can be particularly arduous [65]. Transboundary conservation will require support from the highest levels of government and will be successful when there are overarching legal or coordinating measures to ensure consistency between States [66].

### Conclusions

Among the proposed Mediterranean plans, the EBSAs have political recognition from the Mediterranean States and are contextualized within a global governance mandate of the CBD and UNEPs regional Mediterranean Action Plan. These areas have been defined and recognized by the Parties to the Barcelona Convention and provide a framework to further develop conservation of these priority regions.

Comparison of the priority conservation areas that represent consensus among the multiple initiatives (Figs. 5 and 6) with the EBSAs (Fig. 3G) provides three important insights. First, the consensus areas and the EBSAs largely overlap, indicating that the EBSAs provide a robust synthesis of the varying criteria and data used in different proposals. Therefore, these areas are clear opportunities for conservation action and success in the Mediterranean Sea. Second, the consensus areas are smaller than the EBSAs. Hence the consensus areas can help to identify boundaries and priority areas within the broader regions defined by the EBSAs. Third, the overlap of multiple proposals identified as a top priority additional areas not included in the EBSAs: portions of the southern and eastern Aegean Sea, portions of the central Adriatic Sea and several coastal areas (Figs. 5 and 6). Thus our approach has identified possible gaps in the accepted EBSA proposal for conservation priority areas.

Both within EBSAs and in those areas that have been identified as priorities but that lie outside EBSAs, unilateral and bilateral conservation agreements will be required. The definition of these agreements would be best served through other overarching legal and coordinating measures. For instance, those areas that may extend over two countries that are in the European Union (e.g. for the central Adriatic consensus area, Figure 5, Italy and Croatia, which will join the EU in July 2013) there may be an opportunity to utilize the European directives. Similarly where countries are Parties to the Bern convention (Council Decision 82/72/EEC) there may be an opportunity to develop mechanisms for cooperation through that platform; this could be applied to the Eastern Aegean Sea area shared between Greece and Turkey.

## Supporting Information

Text S1 **Existing conservation areas and proposed priority areas for conservation in the Mediterranean Sea.**
(DOCX)
