# Supplementary material for: Setting Priorities for Regional Conservation Planning in the Mediterranean Sea
Source: PLoS One. 2013 Apr 5;8(4):e59038. doi: 10.1371/journal.pone.0059038 (PMC3618442; doi:10.1371/journal.pone.0059038)
Supplement: Text S1 — Existing conservation areas and proposed priority areas for conservation in the Mediterranean Sea. (DOCX) [file pone.0059038.s001.docx]

**Existing conservation areas in the Mediterranean Sea**

**National initiatives/ Present Marine Protected Areas (MPAs)**

The first MPA in the Mediterranean was established at the beginning of the 1960s. Currently, more than 100 MPAs (Portman et al. in press) have been designated in the Mediterranean Sea, covering 97,410 km² or approximately 4% of its total surface (Abdulla et al. 2008). Excluding the Pelagos Sanctuary (87,500 km²), the area covered by coastal MPAs amounts to only 9,910 km², which is 0.4% of the total surface of the Mediterranean Sea. Cumulative no-take area that has been reported is 202 km², or 0.01% of its total surface. All MPAs are coastal and designated at national or sub-national level (for example, by autonomic governments within countries), except for the high-sea international MPA Pelagos Sanctuary (agreement between 3 countries: France, Monaco and Italy). Current Mediterranean MPAs have been established based more on the presence of charismatic species (e.g. monk seals, gorgonians, red coral, marine turtles) and unique features or opportunity, than on a holistic ecological approach (Abdulla et al. 2009). Finally, most of the MPAs are situated in the north-western Mediterranean while they are almost absent from the southern and south-eastern part of the Basin.

**European Union (EU)/ Nationally Designated Areas (CDDA)**

The European inventory of nationally designated areas holds information about protected sites and about the national legislative instruments, which directly or indirectly create protected areas. The inventory began under the CORINE programme. It is now maintained for EEA by the European Topic Centre on Biological Diversity and is annually updated through Eionet. EEA provides the European inventory of nationally designated areas to the World Database of Protected Areas (WDPA) and to Eurostat. The nationally designated areas data can also be queried online in the European Nature Information System (EUNIS). The inventory is the national module of the Common Database on Designated Areas (CDDA), which also includes information on areas designated under European Community legislation and areas designated under international conventions. CDDA designation categories are: 1. designation types used with the intention to protect species, habitats and landscapes; 2. statutes under sectorial, legislative and administrative acts providing adequate protection relevant to species and habitat conservation; 3. private statute providing durable protection for species and habitats.

**United Nations Educational, Scientific and Cultural Organization (UNESCO)/ World heritage sites**

The Convention Concerning the Protection of the World Cultural and Natural Heritage was adopted by the General Conference of UNESCO in 1972. The primary mission of the Convention **is to identify and protect the world's natural and cultural heritage considered to be of "outstanding universal value"**. The Convention draws up a list of properties called "the World Heritage List", which is made up of natural, cultural and mixed sites and landscapes. It promotes co-operation among all nations and peoples to contribute effectively to the protection of these important properties. The Convention is governed by the World Heritage Committee supported by the UNESCO World Heritage Centre, the secretariat for the Convention, based at UNESCO headquarters in Paris.

To be included on the World Heritage List (http://whc.unesco.org/en/list), sites must be of outstanding universal value and meet at least one out of ten selection criteria. Six inscription criteria relate to cultural heritage (i–vi) and four relate to natural heritage (vii–x). World Heritage sites nominated for natural values need to comply with at least one of the natural criteria:

vii. Contain superlative natural phenomena or areas of exceptional natural beauty and aesthetic importance;

viii. Be outstanding examples representing major stages of Earth’s history, including the record of life, significant ongoing geological processes in the development of landforms, or significant geomorphic or physiographic features;

ix. Be outstanding examples representing significant **ongoing ecological and biological processes** in the evolution and development of terrestrial, fresh water, coastal and marine ecosystems and communities of plants and animals;

x. **Contain the most important and significant natural habitats for in situ conservation of biological diversity, including those containing threatened species of outstanding universal value** from the point of view of science or conservation

In the Mediterranean Sea there are three World Heritage sites that include marine areas. The Ichkeul National Park in Tunisia was inscribed as a World Heritage Site in 1980 and consists of a lake-salt marsh system, which (especially the ense Potamogeton beds) supports huge populations of a few species of marine and brackish water invertebrates. The Gulf of Porto: Calanche of Piana, Gulf of Girolata, Scandola Reserve (Corsica, France) was declared a World Heritage Site in 1983. Seagulls, cormorants and sea eagles can be found there. The clear waters, with their islets and inaccessible caves, host a rich marine life, including considerable numbers of spiny lobster and a wide range of littoral and sublittoral invertebrates and fishes. In 1999, Ibiza Island (Spain) Biodiversity and culture, was also inscribed. The evolution of Ibiza's shoreline is one of the best examples of the influence of the seagrass *Posidonia* on the interaction of coastal and marine ecosystems. The well-preserved *Posidonia* seagrass beds, threatened in most Western Mediterranean locations, contain and support a diversity of marine life.

**Barcelona Convention SPA-BD protocol/ Specially Protected Areas of Mediterranean Importance (SPAMIs)**

To promote **the protection of threatened species and their habitats in the Mediterranean Sea**, and the cooperation in the management and conservation of natural areas, the Contracting Parties to the Barcelona Convention established the List of Specially Protected Areas of Mediterranean Importance (SPAMI's List), through the Protocol concerning Specially Protected Areas and Biological Diversity in the Mediterranean (SPA/BD 1995). According to the SPA/BD Protocol, **SPAMIs may be established in the marine and coastal zones subject to the sovereignty or jurisdiction of the Parties but also in areas situated partly or entirely on the high sea**.

According to Article 8(2) of te SPA/BD Protocol, the SPAMI's List may include sites which: **are of importance for conserving the components of biological diversity in the Mediterranean; contain ecosystems specific to the Mediterranean area or the habitats of endangered species**; are of special interest at scientific, aesthetic, cultural or educational levels.

The SPA/BD Protocol provides the criteria for the choice of protected marine and coastal areas that could be included in the SPAMI's List (Annex I of the SPA/BD Protocol) as well as the procedure and the stages to be followed with the view of including an area in the List. The stated criteria for the evaluation of candidate sites include: (i) **Uniqueness**: the area contains unique or rare ecosystems, or rare or endemic species; (ii) **Natural representativeness**: the area has highly representative ecological processes, or community or habitat types or other natural characteristics; representativeness is the degree to which an area represents a habitat type, ecological process, biological community, physiographic feature or other natural characteristic; (iii) **Diversity**: the area has a high diversity of species, communities, habitats or ecosystems; (iv) **Naturalness**: The area has a high degree of naturalness as a result of the lack or low level of human-induced disturbance and degradation; (v) **Presence of habitats that are critical to endangered, threatened or endemic species**; (vi) **Cultural representativeness**: the area has a high representative value with respect to the cultural heritage, due to the existence of environmentally sound traditional activities integrated with nature which support the well-being of local populations.

The Regional Activity Centre for Specially Protected Areas (RAC/SPA), based in Tunis, was established by the Contracting Parties to the Barcelona Convention and its Protocols in order to assist Mediterranean countries in implementing the SPA/BD. RAC/SPA formulates recommendations for guidelines and common criteria for the selection of marine and coastal protected areas that could be included on the SPAMI List, common criteria for the inclusion of additional species in Annexes II and III to the SPA/BD Protocol, guidelines for the establishment and management of protected areas and any other technical tool relevant to the implementation of the SPA/BD Protocol.

To be included in the SPAMI List, a protected area: (i) must have a management body, endowed with sufficient powers as well as means and human resources to prevent and/or control activities likely to be contrary to the aims of the protected area; (ii) will have to be endowed with a management plan, whose rules are to be laid down as from the time of inclusion and implemented immediately; (iii) will have to be endowed with a monitoring programme.

Currently, the SPAMI List includes **32 sites** (see: http://www.rac-spa.org/spami), among which the Pelagos Sanctuary for marine mammals.

**European Union (EU)/ Natura 2000 Network – Emerald Network**

Two major drivers of establishment of protected areas in the territories of EU Member States are the **Birds Directive** (EC, 2009) and the **Habitats Directive** (EC, 1992). Together these Directives serve as a legally binding basis for the establishment of a set of terrestrial and marine protected areas, collectively known as **Natura 2000** network.

The Birds Directive (EC, 2009) constitutes the **legal framework** established by EU to meet its obligations for bird species conservation under the **Bern and Bonn Conventions**, i.e. providing a framework for the conservation and management of, and human interactions with, wild birds in Europe. Among the measures included in the Birds Directive is the creation of Special Protection Areas (**SPAs**), which include marine areas that serve as habitat for seabirds during all or parts of the year.

The Habitats Directive was established in 1992 by the European Community for the conservation of natural habitats and of wild fauna and flora (EC, 1992), in order to meet the obligations of the Convention on the Conservation of European Wildlife and Natural Habitats (**Bern Convention**). The high level objectives of the Habitats Directive are to ‘...ensure biodiversity through the conservation of natural habitats and wild fauna and flora and to maintain and restore natural habitats and species of wild fauna and flora of community interest.’ In order to reach the objectives set out in the Habitats directive, EU adopted a schedule organized into three phases: In the first phase, each member state was enquired to draw up national lists of sites, based on ecological criteria defined at the European level. **The initial list of sites proposed by each member state was basically expert-driven** (Jongman, 1995; Maiorano et al., 2007). Then, in agreement with each member state and on the basis of proposals received, the Commission established a draft list of Sites of Community Importance (**SCIs**). Finally, SCIs were designated by each member state as Special Areas of Conservation (**SACs**). **SPAs and SACs together constitute the Natura 2000 network.** Marine Natura 2000 sites are in many aspects synonymous with the traditional concept of marine protected areas (MPAs).

Potential SACs were selected based on a list of habitats and species included in Annexes I and II. Currently (after some amendments in 2006), Annex I contains 218 habitats of conservation importance, and Annex II contains 887 species. Of these, **only a small fraction includes marine species and habitats.** There are only **9 Annex I marine habitats** included in Annex I (habitat code in parenthesis): sandbanks which are slightly covered by sea water all the time (1110); *Posidonia oceanica* beds (1120); estuaries (1130); mudflats and sandflats not covered by seawater at low tide (1140); coastal lagoons (1150); large shallow inlets and bays (1160); reefs (1170); submarine structures made by leaking gases (1180); and submerged or partially submerged sea caves (8330). There are only **10 Annex II species** that are relevant for the Mediterranean Sea (species code in parenthesis): the monk seal ***Monachus monachus*** (1366), the harbour porpoise ***Phocoena phocoena*** (1351), the bottlenose dolphin ***Tursiops truncatus*** (1349), the loggerhead turtle ***Caretta caretta*** (1224), the green turtle ***Chelonia mydas*** (1227), the gastropod ***Gibbula nivosa*** (2578), the sea lamprey ***Petromyzon marinus*** (1095), the river lamprey ***Lampetra fluviatilis*** (1099), the allis shad ***Alosa alosa*** (1102), and the twaite shad ***Alosa fallax*** (1103).

The **criteria** for selecting sites eligible for identification as SCIs and designation as SACs are listed in Annex III of the directive:

For habitats (Annex I):

1. The **degree of representativeness** of the natural habitat type on the site. This is the degree to which a habitat corresponds to its described type.

2. **Area of the site** covered by the natural habitat type in relation to the total area covered by that natural habitat type within national territory. Examples with the largest surface area are most desirable.

3. **Degree of conservation** of the structure and functions of the natural habitat type concerned and restoration possibilities. Structure relates to biotic and abiotic features such as species composition and ground morphology whilst function relates to how these features interact over time. The closest to typical structure and function is an example, the most desirable it is.

4. **Global assessment of the value of the site** for conservation of the natural habitat type concerned. This provides an integrated assessment of the other selection criteria and is a judgement of the overall value of the site for the conservation of the relevant Annex I habitat.

For species (Annex II):

1. **Size and density of the population** of the species present on the site in relation to the populations present within national territory. The biggest and most dense populations are most desirable for protection.

2. **Degree of conservation of the habitat features**, which are important for the species concerned and restoration possibilities. Areas that have the best surrounding habitats for the species survival are regarded as the highest quality sites.

3. **Degree of isolation** of the population present on the site in relation to the natural range of the species. Only isolated populations that have a high density recorded over a number of years were considered.

4. **Global assessment of the value of the site** for conservation of the species concerned.

The **Natura 2000 Network together with the Emerald Network** constitute the main components of the **Pan European Ecological Network** (PEEN) which contains key elements from all of the regionally applicable international conservation instruments (Council of Europe, 2011). In 1989 the Standing Committee of the Bern Convention adopted Resolutions for the definition of conservation habitats, and operative recommendations to create protected areas and a protected area network for the region. Recommendation No. 16 (1989) recommends Parties to "take steps to designate **Areas of Special Conservation Interest** (ASCIs) to ensure that the necessary and appropriate conservation measures are taken for each area situated within their territory or under their responsibility where that area fits one or several of the following conditions ":

1. it contributes substantially to the survival of threatened species, endemic species, or any species listed in Appendices I and II of the convention;
2. it supports significant numbers of species in an area of high species diversity or supports important populations of one or more species;
3. it contains an important and/or representative sample of endangered habitat types;
4. it contains an outstanding example of a particular habitat type or a mosaic of different habitat types;
5. it represents an important area for one or more migratory species;
6. it otherwise contributes substantially to the achievement of the objectives of the convention.

In January 1996 Resolution No. 3 (1996) to "set up a network (Emerald Network) which would include the ASCIs designated following Recommendation No. 16 (1989)" was adopted. Recommendation No. 16 (1989) and Resolution No. 3 (1996) oblige Parties to protect the habitats of species and endangered natural habitats as strict obligations clearly marked in the Convention forming part of international law. Thirty-nine marine habitats are listed under Annex I (1996, adopted 2010) and numerous plant and animal species important to the Mediterranean are listed as “species requiring specific habitat conservation measures”.

The Bern Convention (1979) and the Habitats Directive (1992) have coordinating objectives. For Member States of the EU, Resolution No 5 (1998) stipulates that “for contracting parties which are Member States of the European Union **Emerald Network sites are those of the Natura 2000**”. In this respect, the implementation of the Habitats Directive is a fundamental step to achieve the common goals it shares with the Bern Convention. **Member States of the EU will satisfy the habitat requirements of the Bern Convention through the designation of sites to the Natura 2000 Network**. Thus, the Special Areas of Conservation of the Natura 2000 Network will also become ASCIs of the Emerald Network as it is foreseen in Resolution No. 5. This ensures the coherence of the Network for the whole of Europe.

**Ramsar, Convention on Wetlands**

The Convention on Wetlands of 1971, called the "Ramsar Convention", is an intergovernmental treaty that embodies the commitments of its member countries to maintain the ecological character of their Wetlands of International Importance and to plan for the "wise use", or sustainable use, of all of the wetlands in their territories.

As defined by the Convention, wetlands include a wide variety of habitats such as **marshes, peatlands, floodplains, rivers and lakes, and coastal areas such as saltmarshes, mangroves, and seagrass beds, but also coral reefs and other marine areas no deeper than six metres at low tide**, as well as human-made wetlands such as waste-water treatment ponds and reservoirs.

The designation of Wetlands of International Importance is based on 9 criteria:

**Criterion 1**: Representativeness, rarity, or uniqueness of a natural or near-natural wetland type found within the appropriate biogeographic region.

**Criterion 2**: Inclusion of vulnerable, endangered, or critically endangered species or threatened ecological communities.

**Criterion 3**: Support of populations of plant and/or animal species important for maintaining the biological diversity of a particular biogeographic region.

**Criterion 4**: Support of plant and/or animal species at a critical stage in their life cycles, or provision of refuge during adverse conditions.

**Criterion 5**: Regular support of 20 000 or more waterbirds.

**Criterion 6**: Regular support of 1% of the individuals in a population of one species or subspecies of waterbirds.

**Criterion 7**: Support of a significant proportion of indigenous fish subspecies, species or populations that are representative of wetland benefits and/or values and thereby contributes to global biodiversity.

**Criterion 8**: Important source of food for fishes, spawning ground, nursery and/or migration path on which fish stocks, either within the wetland or elsewhere, depend.

**Criterion 9**: Regular support of 1% of the individuals in a population of one species or subspecies of wetland-dependent non-avian animal species.

Having systematically applied the Criteria to develop a list of wetlands that qualify for designation, Contracting Parties are encouraged to identify priority candidate sites. Ramsar guidelines dictate that particular weight should be given to designating sites which include wetland types, or wetland species, that are either unique/endemic to the Contracting Party (found nowhere else in the world) or for which that country holds a significant proportion of the total global extent of a wetland type or population of a wetland species. In the Mediterranean, there are currently more than 100 Ramsar sites that include coastal areas.

**Proposed priority areas for conservation in the Mediterranean Sea**

**World Wildlife Fund Mediterranean Programme Office (WWF MedPO)/ 13 key Mediterranean marine areas in need of protection**

In 1998, the Mediterranean Programme Office of the World Wide Fund for Nature (WWF MedPO) conducted a Mediterranean Marine Gap Analysis, which identified **13 key marine and coastal areas for biodiversity** in the Mediterranean where there is an urgent need for new reserves and changes in coastal management practices.

In order to identify **rich biodiversity regions,** statistical approaches were used to analyse the “indentation” of coasts and the “roughness” of the sea-bottom, enabling the visualisation of areas that were more likely to have higher levels of biodiversity within a depth range of 0 and 250 m (considering that 80% of marine biodiversity can be found within this range). Additionally existing data on the **presence of flagship species** such as monk seal, marine turtles, several species of whales and dolphins and the sea grass *Posidonia oceanic* were mapped. The main coastal **pollution “hot spots”**, identified by UNEP-MAP, were also included in the analysis. The **human impact on the coast** was also mapped, taking into consideration of main harbours and coastal cities with more than 50 000 inhabitants.

Through the overlapping of the data layers, described above, the most significant marine conservation areas for the Mediterranean Sea were identified. The **13 areas** in need of urgent protection or improved management are: 1. **Alboran Sea** (Spain, Morocco, Algeria), 2. **Balearic Islands** (Spain), 3. **Liguro-Provencal coast** (France, Italy, Monaco), 4. **Corso-Sardinian coast** (France, Italy), 5. **Southern Tyrrhenian coast** (Italy), 6. **Dalmatian coast** (Croatia), 7. **Eastern Ionian coast and islands** (Albania, Greece), 8. **Aegean Sea and Anatolia coast** (Greece, Turkey), 9. **Cicilian coast** (Turkey) and **Cyprus Island coast**, 10. **Cyrenaica** (Libya), 11. **Culf of Sirte** (Libya), 12. **Gulf of Gabes** (Tunisia) and 13. **Algero-Tunisian coast** (Algeria, Tunisia).

**General Fisheries Commission for the Mediterranean (GFCM)/ Fisheries Restricted Areas**

The objectives of the Agreement establishing the General Fisheries Commission for the Mediterranean (GFCM) are **to promote the development, conservation, rational management and proper utilisation of living marine resources**. In order to **protect the structure and functioning of marine ecosystems** and **benefit sustainable exploitation of marine resources**, GFCM has proposed through various Recommendations the restriction of specific fishing gears in different areas of the Mediterranean. These areas are known as FRAs (Fisheries Restricted Areas).

GFCM/2006/3 Recommendation on the “establishment of fisheries restricted areas in order **to protect the deep sea sensitive habitats**” dictates that fishing with towed dredges and bottom trawl nets shall be prohibited in the following three areas: the Lophelia Reefs of Santa Maria di Leuca; the area of cold upwellings of the Nile Delta; and the Eratosthenes Seamount. Members of GFCM are asked to call the attention of the appropriate authorities in order to protect these areas from the impact of any other activity jeopardizing the conservation of the features that characterize these particular habitats.

GFCM/33/2009/1 Recommendation on the “establishment of a fisheries restricted area in the Gulf of Lions **to protect spawning aggregations and deep sea sensitive habitats**” adopted a FRA in the eastern Gulf of Lions, where: the fishing effort for demersal stocks of vessels using towed nets, bottom and mid-water longlines, and bottom-set nets shall not exceed the level of fishing effort applied in 2008; Members and cooperating non-Members of GFCM shall create registers of fishing vessels authorized to fish in the area and establish legal restrictions as for the maximum time of daily fishing activity and the maximum number of days a vessel can stay within the FRA; Members of GFCM shall call the attention of the appropriate national and international authorities in order to protect this area from the impact of any other human activity jeopardizing the conservation of the features that characterize this particular habitat as an area of spawners’ aggregation.

In addition to the establishment of the above FRAs, GFCM recommended the prohibition of towed dredges and trawl nets fisheries at depths beyond 1000m (Recommendation GFCM/2005/1 on the “management of certain fisheries exploiting demersal and deepwater species”). The EU has adopted this recommendation and prohibited the use of towed dredges and trawlers at depths beyond 1,000 m (EC, 2006), which potentially protects an important part of the Mediterranean deep-sea ecosystems, such as **deep-sea corals** (EUNIS A6.61), **deep-sea sponge aggregations** (EUNIS A6.62), **seamounts** (EUNIS A6.72), **oceanic ridges** (EUNIS A6.73), and **submarine canyons on the continental slope** (EUNIS A6.81) (Salomidi et al. 2012), and deep-sea organisms (Cartes et al. 2004).

**GREENPEACE/ Marine reserves for the Mediterranean Sea**

The Greenpeace proposal for a regional network of marine reserves was based on data relating to the **biological diversity** and **physical oceanography** of the Mediterranean. Once collected, the data (mainly spatial, but some quantitative) was digitised and inputted into a Geographical Information System (**GIS**) database. Then areas of high ecological importance were determined. **At least 40% of each habitat** was included.

Data layers used in mapping the network included:

• Distribution of species (including **whales, dolphins, seals and fish**)

• Important areas for marine species (such as **spawning grounds**, **nursery areas** and **nesting beaches**)

• Important habitats (such as **seamounts** and **seagrass meadows**)

• Sites previously identified as priorities for protection (such as **SPAMI** and **Natura**

**2000 sites**)

To further improve the process, **expert advice** from regionally based scientists was sought.

**Agreement on the Conservation of Cetaceans in the Black Sea, Mediterranean Sea and the Contiguous Atlantic Area (ACCOBAMS)/** **Existing and proposed MPAs for whales and dolphins in the Mediterranean and the Black Seas**

In order to identify **priority areas for the conservation of cetaceans** in the Mediterranean and Black Seas, the Agreement on the Conservation of Cetaceans in the Black Sea, Mediterranean Sea and the Contiguous Atlantic Area (ACCOBAMS) **requested from its members to submit proposals of potential sites**. In those proposals, criteria for the selection of critical habitats for cetaceans included:

1. Areas used by cetaceans for feeding, breeding, calving, nursing and social behavior;
2. Migration routes and corridors and related resting areas;
3. Areas where there are seasonal concentrations of cetacean species;
4. Areas of importance to cetacean prey;
5. Natural processes that support continued productivity of cetacean foraging species (upwellings, fronts, etc.);
6. Topographic structures favourable for enhancing foraging opportunities for cetacean species (canyons, seamounts).

Furthermore, **anthropogenic threats** to critical cetacean habitats were considered:

1. Conflicts between cetaceans and fishing activities;
2. Significant or frequent bycatch of cetaceans;
3. Intensive whale watching or other marine tourism activities;
4. Navigation;
5. Pollution runoff, outflow or other marine dumping;
6. Military exercises.

Then the Meeting of the Parties to ACCOBAMS, taking into consideration the recommendation of the Scientific Committee, proposed a plan of **22 Marine Protected Areas for the conservation of cetaceans in the Mediterranean and Black Seas**.

**Vulnerable Habitats (De Juan and Lleonart, 2010)**

De Juan and Lleonart (2010) identified **12 priority conservation areas**, which are **vulnerable to fishing activity** and should be protected to avoid their degradation. This initiative emerged within the framework of the Mediterranean Group of the Scientific, Technical and Economic Committee for Fisheries (STEFCF/SGMED) of the European Commission, during its meeting in Rome between 6-10 March 2006 (SGMED 2006). The main aim of the working group was to identify and map sensitive and essential marine habitats in the Mediterranean Sea that are crucial for the conservation of fish and shellfish resources. The identified areas by de Juan and Lleonart (2010) are largely equivalent to the Ecologically or Biologically Significant Areas (EBSAs) described in the criteria adopted by the Convention on Biological Diversity.

Two levels of vulnerable habitats were defined: **Essential Fish Habitats** (EFH) and **Sensitive Habitats** (SH). According to STECF: EFH is a habitat identified as essential for the ecological and biological requirements for critical life history stages of exploited fish species, and may require special protection to improve stock status and long term sustainability; SH are fragile habitats that are recognized internationally as ecologically important and which support important assemblages of commercial and non-commercial fish species and may require special protection.

De Juan and Lleonart (2010) focused on those high seas habitats considered as “emblematic” and attempted to encompass a wide range of sites covering the diversity of habitats in the Mediterranean. These sites were identified based on criteria of uniqueness or rarity; special importance for life history stages of species; importance for threatened, endangered or declining species and/or habitats; vulnerability to fishing and slow recovery after disturbance; biological productivity; biological diversity and naturalness.

The proposed areas included **spawning grounds and nursery areas for demersal and pelagic commercial species** such as hake (***Merluccius merluccius***), rose shrimp (***Parapenaeus longirostris***), red mullet (***Mullus barbatus***), monkfish (***Lophius* spp.**), blue and red shrimp (***Aristeus* antennatus**), Norway lobster (***Nephrops norvegicus***), bluefin tuna (***Thunnus thynnus***), albacore (***Thunnus alalunga***), and large organisms such as sperm whales (***Physeter macrocephalus***), white shark (***Carcharodon carcharias***), and **migratory routes for many species of tuna, whales, dolphins and turtles**. Sensitive habitats considered included: **deep‐water corals, seamounts, canyons, and cold hydrocarbon seeps**.

**United Nations Environment Programme Mediterranean Action Plan,** **Regional Activity Centre for Specially Protected Areas** (**UNEP MAP, RAC-SPA)/ Important seabirds areas**

This initiative represents a first step towards the selection of protected areas for seabirds at pan-Mediterranean scale and has been developed in the framework of the activities of the UNEP Regional Activity Centre for Specially Protected Areas (RAC/SPA) in compliance with the Mediterranean SPA/BD Protocol.

**Pelagic Mediterranean endemic and near-endemic seabird species** known to occur regularly in offshore waters were considered: Cory’s Shearwater (*Calonectris diomeda diomeda*), Yekouan Shearwater (*Puffinus yelkouan*), Balearic Shearwater (*Puffinus mauretanicus*), European Storm-Petrel (*Hydrobates pelagicus melitensis*), Mediterranean Shag (*Phalacrocorax aristotelis desmarestii*), Mediterranean Gull (*Larus melanocephalus*) and Audouin’s Gull (*Larus audouinii*).

Species' known distribution (both breeding and wintering ranges) were obtained from various sources: published references, consultation to experts, data from censuses at sea and satellite telemetry, plus authors’ data. The information was compiled and mapped in GIS format in a 10x10-km grid following the recommendations of the EEA. The resulting cells were assigned a numerical value based on the number of species present and their conservation value (endemic and/or in decline). Treatment of the data allowed for graphic representation and for some geographical calculations, as well as for further analysis along with environmental variables: bathymetry, shoreline, sea surface temperature, sea productivity (Chlo-a) and seamount proximity. When all data layers were superimposed, all the cells were ranked between 0 and 7, according to the sum of species contained and their vulnarability.

The distribution of priority B areas marks the influence of large-scale ocean graphical features (increased productivity, mixing of waters, bathymetric zone) and acts as a general indicator of areas of conservation interest for seabirds. Given the paucity of data for some species and regions, priority B areas should be taken as ‘good areas’ for the development of a network of marine protected areas for the conservation of seabirds. Nearly one-fourth (25.75%) of the sum of cells corresponded to cat. 2-4; these were assigned ‘priority B’.

Priority A areas are found within the limits of priority B zones in all cases. Characteristically, they are found on the continental shelf, around breeding islands or where key oceanographic features (fronts, upwellings) occur, also, outstandingly, in the Straits of Gibraltar. Less than 10% (8.88%) of the total sum of cells had the highest importance (cat. 5-7) in terms of priority bird species present; these were assigned ‘priority A’.

The following sites were identified as areas of specific ornithological importance: **straits of Gibraltar, Malaga coast, Gulf of Almeria, Central Alboran sea, Morocco-Algerian coast, Almeria – Oran front, Gulf of Lions, Iles d’Hyeres, Cap de Creus coast, Ebro delta & river system, Columbretes Islands, Balearic Islands, Cap de la Nau, Tuscan coasts and islands, N & E Corsica, Corsican-Sardinian islands & shelf, NW Sardinia, Lazio coast, Aeolian islands, Strait of Sicily-Tunisia / Cap Bon Channel, Egadi Islands, Pantelleria & Pelagie Islands, Malta and Sicilian waters, Tunisian-Lybian waters, Nile river system, Levantine coast, Southern Aegean Sea – Sea of Crete, Northern Aegean Sea – Thracian Sea, Sea of Marmara, Bosporus and Dardanelles, Gulf of Venice, and Central Adriatic Sea**.

**UNEP MAP, RAC-SPA and EU/ Ecologically or Biologically Significant Areas (EBSAs)**

The United Nations Environment Programme - Mediterranean Action Plan (UNEP-MAP) and the European Commission (EC) financed the effort to create a network of Specially Protected Areas of Mediterranean Importance (SPAMIs) in the Mediterranean open seas, including the deep seas. The work involved a three-step hierarchical planning approach, beginning with the subdivision of the Mediterranean in eight large ecological units: 1. Alboran Sea, 2. Algero-Provencal Basin, 3. Tyrrhenian Sea, 4. Adriatic Sea, 5. Tunisian Plateau/Gulf of Sidra, 6. Ionian Sea, 7. Aegean Sea, and 8. Levantine Sea.

At the next step, Ecologically or Biologically Significant Areas (EBSAs) were identified within these subregions by Notarbartolo di Sciara and Agardy (UNEP(DEPI)/MED WG.348/Inf.3). These areas were not MPAs themselves, but were focal areas for individual MPA networks. The EBSAs were selected according to seven criteria, which have been proposed within the Convention on Biological Diversity (CBD): uniqueness or rarity; special importance for life history stages of species; importance for threatened, endangered or declining species and/or habitats; vulnerability, fragility, sensitivity or slow recovery; biological productivity; biodiversity; and naturalness. Experts of Mediterranean marine ecology, biodiversity, oceanography and geomorphology, defined 90 polygons in all 8 Mediterranean sub-regions. Experts were then asked to score polygons on the basis of the 7 CBD criteria. Polygon types included: **known habitats of cetaceans, monks seals, marine birds, marine turtles, sharks and bluefin tuna (59); geological features such as canyons, seamounts, other significant seafloor geomorphological features. (18); high primary productivity areas (either permanent or seasonal) and known locations of deep-sea coral reefs (*Lophelia* and *Madrepora*)**.

Complementary thematic studies were conducted to complete this assessment for EBSA identification (UNEP-MAP-RAC/SPA, 2012):

- **deep-sea fauna and locations important for fisheries** were addressed

- **bird important areas** were mapped

The ‘Fisheries conservation management and vulnerable ecosystems in the Mediterranean open seas, including deep seas’ study (UNEP(DEPI)/MED WG.348/Inf.4) enabled areas that contain vulnerable ecosystems to be identified on the basis of data relative to fisheries and their impact on ecosystems and species, according to:

- the **geological features** of the seabed (mud volcanoes, seamounts, dries, canyons, hydrothermal vents; these areas have a high rate of endemism)

- **oceanographic features** such as upwellings (characterised by high productivity)

- the **ecological features of certain habitats** (coralligenous facies, white coral communities)

- the biogeographic features of commercial pelagic species and species subject to incidental capture or by-catch (**spawning and nursery areas**).

Also, the ‘Georeferenced compilation on bird important areas in the Mediterranean Open Seas’ study (UNEP(DEPI)/MED WG.348/Inf.5) enabled the mapping of the distribution of threatened or endangered pelagic marine bird species. This study was mostly based on seven out of the fifteen bird species listed in Annex II to the SPA/BD Protocol, specifically *Calonectris diomedea diomedea* (Mediterranean subspecies), *Puffinus yelkouan* (endemic), *Puffinus mauretanicus* (endemic), *Hydrobates pelagicus melitensis* (Mediterranean subspecies), *Phalacrocorax aristotelis desmarestii* (Mediterranean subspecies), *Larus audouinii* (endemic), *Larus melanocephalus* (near-endemic). These seven species being found far out from the coast are more representative of the pelagic habitats, have high levels of endemism, and high levels of threat.

To facilitate the cross analysis of all this data, the different layers from the different studies were compiled in a Geographical Information System producing the “Technical report on the geographical information system developed for Mediterranean open seas” (UNEP-MAP-RAC/SPA, 2010).

The 12 EBSAs identified were: 1. Alboran seamounts; 2. Southern Balearic; 3. Gulf of Lions shelf and slope; 4. Central Tyrrhenian Sea; 5. Northern strait of Sicily (including Adventure and nearby banks); 6. Southern strait of Sicity (Tunisian plateau); 7. Northern and central Adriatic; 8. Santa Marina di Leuca; 9. North-eastern Ionian Sea; 10. Thracian Sea; 11. North-eastern Levantine Sea and Rhodos gyre; and 12. Nile Delta Region.

Initially, Eratosthenes seamount was also included in the list of EBSAs but it was later removed after the objection of Cyprus.

**The Mediterranean Science Commission (CIESM)/ Mediterranean Marine Peace Parks**

CIESM (*Commission Internationale pour l'Exploration Scientifique de la mer Méditerranée*), founded by Albert I of Monaco, is an intergovernmental body with 23 member states around the Mediterranean coast, aiming to promote multilateral international research on marine science. The main goal of CIESM workshop n. 41 held in Syracuse in November 2010 (CIESM, 2011) was to define vast areas for conservation, straddling both open sea and coastal waters, in order to effectively preserve Mediterranean biodiversity and the essential services it provides, through the creation of cross-border marine parks. Twenty-eight invited experts in the Mediterranean region identified and provided all available information for **large, coast-to-coast, trans-frontier priority marine areas. These areas enclosed key hydrodynamic, biological and geological features**. The intention of this initiative is to protect more than 10% of the Mediterranean Sea before 2020.

Based on the criteria set, eight large marine areas have been selected: the Pelagian Sea (area between Tunis and Sicily), the Strait of Gibraltar *sensu lato*, sectors of the Ionian, Adriatic, north Levantine and south Aegean Seas. The underlying idea of **‘peace parks’** (Sandwith et al. 2001) is that such trans-boundary protected areas would improve existing relations between neighbouring States, and render current disputes over the precise demarcation of maritime frontiers less and less relevant.

Marine Peace Parks aimed to **preserve endemic, threatened biota** (unique deep-sea communities, white coral beds, monk seals, rare endemic species, fin whales, spawning grounds of bluefin tuna, etc.), but also **unique geological features** (deep sea canyons, mud volcanoes, seamounts, hypersaline craters), and **key oceanographic processes** (surface sites of deep water formation, strait fluxes) (CIESM, 2011). They also aimed to integrate contiguous coastal and open sea habitats, providing suitable range and connecting corridors to larger animals that actively move across the seascape, enhancing the robustness and resilience of the ecosystem in the face of climate change, and thus ultimately enhancing resource yields and local economies (CIESM, 2011).

**No specific boundaries** or a zoning plan were given for the eight proposed marine areas. Their selection was based on **expert opinion** and was justified by reviewing available information and knowledge on the key geological, oceanographic, and biological features of the proposed areas, including general information on the biodiversity, presence of endangered/key species, critical habitats and unique communities/ecosystems, and main threats.

**OCEANA/ MedNet: MPA network proposal for the Mediterranean Sea**

Biological data compiled by Oceana was used as background data. Other sources used included information about underwater relief from GEBCO (General Bathymetric Chart of the Oceans) and the potential locations of seamounts as reported by Kitchingman et al. (2007). All the locations with the above mentioned features were compiled in a Geographic Information System (**GIS**) and after a process of homogenisation and standardisation, 385 priority sites emerged. The study area was subdivided in eco-regions. The division by the General Fisheries Commission for the Mediterranean (GFCM) was used, slightly modified.

Once all of the locations were obtained all available information was gathered which took into account the initiatives at Mediterranean scale. The tool used for storing all of this information was an Access database. Various criteria were used for the final selection of sites:

● **Biological**: 1. **Key species** (commercial or biological/ecological interest), 2. **CBD criteria**.

● **Geological**: 1. Type of elevation or geological formation (**escarpments, seamounts, canyons, trenches**, etc.).

● **Administrative**: 1. Affected by waters of national jurisdiction and 2. Jurisdictional conflicts

● **Oceanographic**: 1. Connection by currents, gyres and 2. Fronts.

● Detected or potential **threats** 1. Illegal, Unregulated and Unreported Fishing; 2. Potential oil and gas prospecting; 3. Pollution; 4. Maritime traffic; 5. By‑catch.

● **Available scientific documentation** (biological, geological, oceanographic, etc.).

● **Existing proposals**: 1. ACCOBAMS; 2. Barcelona Convention ‑ SPA/BD Protocol; 3. Vulnerable habitats impacted by fishing activities (EFH/SH); 4. GFCM (FRAs); 5. Greenpeace.

Out of this information **159 sites** were initially selected and after an analysis of each site, the different locations were put into groups based on proximity. The final result was 100 priority areas, constituting Oceana´s proposal for a network of Mediterranean MPAs, MedNet. The OCEANA MedNet is mostly comprised of MPAs between 200‐2,300 km², and there are three notable MPAs which are larger than 10,000km² (Mersa‐Matruth Eddy, Malta Ridge and Eratosthenes Seamount).

Recently, out of these 100 sites, Oceana proposed **30 sites** as priority areas which were selected according to the above mentioned criteria (Oceana 2012).

**Cumulative Impact Map (Micheli et al. 2011)**

The aim of this analysis was to quantify and map the cumulative impact of multiple pressures to Mediterranean marine ecosystems and to investigate opportunities for conservation and restoration in the Mediterranean Sea. In particular, the analysis was motivated by the following questions: (1) What are the most and least impacted ecoregions across the Mediterranean basin? (3) What are the top threats affecting the Mediterranean Sea? (4) What areas represent top priorities in terms of need and opportunities for ecosystem-based management and marine spatial planning implementation?

This analysis built on a previous global analysis of **cumulative human impacts** (Halpern et al. 2008). The same approach developed for the global analysis was applied in the Mediterranean, but some of the data layers were replaced and additional data were included to better reflect the specific pressures and ecosystems of the Mediterranean basin. A total of **18 spatial datasets of human activities and stressors** and **17 ecosystem types** were assembled and used in this analysis. Human activities and stressors included: artisanal fishing; coastal population density; demersal, destructive fishing; demersal, non-destructive, high bycatch fishing; demersal, non-destructive, low bycatch fishing; hypoxia; invasive species; nonpoint, inorganic pollutants; nonpoint, organic pollutants; nutrient input; ocean acidification; oil accidents; oil rigs (benthic structures); pelagic, high by-catch fishing; pelagic, low by-catch fishing; pollution from ports; Sea Surface Temperature change; shipping (navigation); urbanization trends; UV change.

The map of cumulative human impacts highlights the widespread distribution of human activities and stressors, and resulting potential impacts throughout the Mediterranean Sea. Areas showing low impact are present within the central Thyrrenian Sea, the Gulf of Sidra, the Adriatic Sea, particularly along the coast of Croatia, the southern Levantine Sea and the Black Sea. Conversely, hot spots of impact were found within the Alboran Sea, the Gulf of Lyons, the Sicily Channel and Tunisian Plateau, the Adriatic Sea, off the coasts of Egypt and Israel, along the coasts of Turkey, and within the Marmara and Black Seas.

More information may be found in: <http://globalmarine.nceas.ucsb.edu/mediterranean/>

**Fish biodiversity (Mouillot et al. 2011)**

Mouillot et al. (2011) adopted a multifaceted approach to biodiversity by considering the species richness of **total, endemic, and threatened (IUCN list) coastal fish assemblages** as well as their **functional and phylogenetic diversity**, showing that these fish biodiversity components are spatially mismatched. Range maps for 282 coastal species, among which 81 are endemic and 45 are on the IUCN Red List, were digitalized on a regular grid covering the continental shelf. Nonnative, migratory large pelagic species as well as those mainly occurring beyond the continental shelf (60% or more of their total range) were also excluded. From a dated phylogeny and a functional dendrogram built using 15 functional traits, phylogenetic and functional diversity of fish assemblages contained in each grid cell were respectively computed using a standardized effect size estimation to provide a diversity value independent of species richness. Then the mapped distribution of species richness, endemic and threatened species, functional diversity and phylogenic diversity were overlaid with the mapped distribution of fishing pressure and existing MPAs identifying hot spots for all fish biodiversity components. In terms of total species richness and IUCN species richness, hotspots were identified off the Spanish, French and Western Italian coasts. Regarding endemic species richness hotspots were identified in the Eastern Adriatic Sea, while hotspots for phylogenetic diversity were located in the south-western part of the Mediterranean Basin (off the coast of Morocco, Algeria and Tunisia). Finally functional diversity hotspots were locate in the Gulf of Gabes (Tunisia) and close to the Nile Delta in Egypt.

**Conservation Concern Areas (Coll et al. 2012)**

Data on species diversity distribution of all **marine mammals, marine turtles, seabirds, fishes and commericially or well-documented invertebrates** in the Mediterranean Sea were collected, mostly in forms of **expert-drawn maps, online databases or sighting locations**. The spatial patterns of invertebrate and vertebrate species were mapped using **GIS** software. Five species groupings were defined and mapped: 1) invertebrates, 2) fishes, 3) marine mammals and turtles, 4) seabirds, and 5) large predators (wich included data from previous 2,3,4 layes).

Data on **18 direct and indirect anthropogenic threats** in the Mediterranean Sea were also collected. Then six layers of potential cumulative threats were created: a. coastal-based impacts (including inorganic pollution, organic pollution, nutrients, hypoxia sites, invasive species, and fish and shellfish aquaculture); b. trawling and dredging disturbance (with benthic disturbance from fishing); c. ocean-based pollution (with ocean-based pollution from shipping traffic, poison shipments, deposition of heavy metals, inorganic nitrogen deposition); d. exploitation of marine resources by fisheries (including demersal and pelagic high and low by-catch and high and low habitat modification, and artisanal fishing); e. maritime activities (with commercial shipping lanes, and benthic oil rig sturctures); and f. climate change (with sea surface temperature anomalies, UV increase and ocean acidification). GIS software was used to map patterns of anthropogenic impacts of each threat to create **cumulative threat layers** (or threat models).

**Spatial distribution of biodiversity groups and cumulative threat layers were combined as to identify priority areas for conservation or “conservation concern” areas: areas where high biodiversity and high cumulative threats occurred simultaneously.** Moreover, the overlap between these priority areas and currently established MPAs was investigated. The overlap found between consrevation concern areas and current MPAs was very small, less than 2%, thus the authors argued that these areas of consrevation concern may be good candidates for future conservation initiatives

**Supplementary References**

Abdulla A, Gomei M, Maison E, Piante C (2008) Status of Marine Protected Areas in the Mediterranean Sea. Malaga: IUCN and France: WWF. 152 p.

Abdulla A, Gomei M, Hyrenbach D, Notarbartolo-di-Sciara G, Agardy T (2009) Challenges facing a network of representative marine protected areas in the Mediterranean: prioritizing the protection of underrepresented habitats. ICES Journal of Marine Science 66: 22-28.

Carboneras C, Requena S (2010) GIS mapping of seabird distribution – a pan-Mediterranean perspective. Poster. 39th Congress of the CIESM, Venice, May 2010.

CIESM (2011) Marine Peace Parks in the Mediterranean – A CIESM proposal. No 41 In: Briand F, editor. Monaco: CIESM Workshop Monographs, 128 p.

Cartes JE, Maynou F, Sarda F, Company JB, Lloris D, et al. (2004) The Mediterranean deep-sea ecosystems: an overview of their diversity, structure, functioning and anthropogenic impacts, with a proposal for their conservation. Malaga: IUCN and Rome: WWF. Part I: pp 9–38.

Coll M, Piroddi C, Albouy C, Ben Rais Lasram F, Cheung WWL, et al. (2012) The Mediterranean under siege: spatial overlap between marine biodiversity, cumulative threats and marine reserves. Global Ecology and Biogeography 21(4): 465-481.

Council of Europe (CoE) (2011) Convention on the Conservation of European Wildlife and Natural Habitats: Group of Expert on Protected Areas and Ecological Networks. The Emerald Network, a network of Areas of Special Conservation Interest for Europe, Information Document, PA07e_2011.doc. 79 p.

De Juan S, Lleonart J (2010) A conceptual framework for the protection of vulnerable habitats impacted by fishing activities in the Mediterranean high seas. Ocean & Coastal Management 53: 717-723

EC (1992) Council Directive on the conservation of natural habitats and of wild fauna and flora. European Commission, Directive 92/43/EEC, OJ L 206.

EC (2006) Council Regulation concerning management measures for the sustainable exploitation of fishery resources in the Mediterranean Sea. Regulation 1967/2006, OJ L 409.

EC (2009) Directive of the European Parliament and the Council on the conservation of wild birds. European Commission, Directive 2009/147/EC, OJ L 20.

EEA (2011) <http://www.eea.europa.eu/data-and-maps/data/nationally-designated-areas-national-cdda-3> Accessed 14 September 2012.

Halpern BS, Waldbridge S, Selkoe KA, Kappel CV, Micheli F, et al. (2008) A global map of human impact on marine ecosystems. Science 319: 948-952.

Jongman RHG (1995) Nature conservation planning in Europe: developing ecological networks. Landscape and Urban Planning 32: 169-183.

Kitchingman A, Lai S, Morato T, Pauly D (2007) How many seamounts are there and where are they located? In: Pitcher TJ, Morato T, Hart P, Clark M, Haggan N, Santo R, editors. Seamounts: Ecology, Fisheries and Conservation. Oxford: Blackwell Fish and Aquatic Resources Series 12. pp 26-40.

Maiorano L, Falcucci A, Garton EO, Boitani L (2007) Contribution of the Natura 2000 network to biodiversity conservation in Italy. Conservation Biology 21: 1433-1444.

Micheli F, Walbridge S, Halpern B (2011) A map of cumulative human impacts on Mediterranean marine ecosystems. <http://globalmarine.nceas.ucsb.edu/mediterranean/> Accessed 14 September 2012.

Mouillot D, Albouy C, Guilhaumon F, Ben Rais Lasram F, Coll M, et al. (2011) Protected and threatened components of fish biodiversity in the Mediterranean Sea. Current Biology 21(12): 1044-1050.

Notarbartolo di Sciara G, Agardy T (2009) Final overview of scientific findings and criteria relevant to identify SPAMIs in the Mediterranean areas beyond national jurisdiction. Second meeting of the Steering Committee on the Identification of Possible SPAMIs in the Mediterranean Areas Beyond National Jurisdiction (ABNJ), Genoa, 18-19 November 2009. Tunis: UNEP MAP, RAC/SPA. 72 p.

Oceana (2011) Oceana MedNet: MPA Network Proposal for the Mediterranean Sea. <http://oceana.org/en/eu/media-reports/publications/mpa-network-proposal-for-the-mediterranean-sea-english-francais>. Accessed 14 September 2012.

Oceana (2012) Oceana MedNet Overview. <http://oceana.org/en/eu/our-work/habitats-protection/mediterranean/mednet/overview>. Accessed 14 September 2012.

Portman ME, Nathan D, Levin N (2012) From the Levant to Gibraltar: A Regional Perspective for Marine Conservation in the Mediterranean Sea. Ambio - Journal of the Human Environment. In press.

Ramsar Convention Secretariat (2010) Designating Ramsar Sites: Strategic Framework and guidelines for the future development of the List of Wetlands of International Importance, Ramsar handbooks for the wise use of wetlands, 4th edition, vol. 17. Gland: Ramsar Convention Secretariat.

Salomidi M, Katsanevakis S, Borja Á, Braeckman U, Damalas D, et al. (2012) Assessment of goods and services, vulnerability, and conservation status of European seabed biotopes: a stepping stone towards ecosystem-based marine spatial management. Mediterranean Marine Science 13(1): 49–88.

Sandwith T, Shine C, Hamilton L, Sheppard D (2001) Transboundary protected areas for peace and co-operation. Gland and Cambridge: IUCN. Xi+111 p.

SGMED (2006) Sensitive and essential fish habitats in the Mediterranean Sea. Scientific, Technical and Economic Committee for Fisheries: Report of the SGMED 06-01 Working Group, Rome, 6-10 March 2006.

SPA/BD (1995) Protocol concerning Specially Protected Areas and Biological Diversity in the Mediterranean. Barcelona Convention, Barcelona, 9-10 June 1995.

UNEP-MAP-RAC/SPA (2010) Report presenting a georeferenced compilation on bird important areas in the Mediterranean open seas. Requena S, Carboneras C, editors. RAC/SPA, Tunis: 39 p.

UNEP-MAP-RAC/SPA (2010) Technical report on the geographical information system developed for Mediterranean open seas. By Requena, S. Ed. RAC/SPA, Tunis: 50pp.

UNEP-MAP-RAC/SPA (2012) Synthesis report on the work carried out regarding EBSAs identification in the Mediterranean. 30pp.

WWF (1998) 13 key Mediterranean marine areas in need of protection.

awsassets.panda.org/downloads/background.doc. Accessed 14 September 2012.
